# Decreased Frequency of Intestinal CD39^+^ γδ^+^ T Cells With Tissue-Resident Memory Phenotype in Inflammatory Bowel Disease

**DOI:** 10.3389/fimmu.2020.567472

**Published:** 2020-09-24

**Authors:** Jana Libera, Melanie Wittner, Marcus Kantowski, Robin Woost, Johanna M. Eberhard, Jocelyn de Heer, Dominik Reher, Samuel Huber, Friedrich Haag, Julian Schulze zur Wiesch

**Affiliations:** ^1^I. Department of Medicine, Infectious Disease Unit, University Medical Center Hamburg-Eppendorf, Hamburg, Germany; ^2^German Center for Infection Research (DZIF), Partner Site Hamburg Lübeck Borstel Riems, Hamburg, Germany; ^3^Clinic and Polyclinic for Interdisciplinary Endoscopy, University Medical Center Hamburg-Eppendorf, Hamburg, Germany; ^4^Institute of Immunology, University Medical Center Hamburg-Eppendorf, Hamburg, Germany

**Keywords:** CD39, CD73, ATP, adenosine, γδ^+^ T cells, gut, IBD, tissue-residency

## Abstract

The ectoenzymes CD39 and CD73 play a major role in controlling tissue inflammation by regulating the balance between adenosine triphosphate (ATP) and adenosine. Still, little is known about the role of these two enzymes and ATP and its metabolites in the pathophysiology of inflammatory bowel disease (IBD). We isolated mononuclear cells from peripheral blood and lamina propria of the large intestine of patients diagnosed with IBD and of healthy volunteers. We then comprehensively analyzed the CD39 and CD73 expression patterns together with markers of activation (HLA-DR, CD38), differentiation (CCR7, CD45RA) and tissue-residency (CD69, CD103, CD49a) on CD4^+^, CD8^+^, γδ^+^ T cells and mucosa-associated invariant T cells using flow cytometry. CD39 expression levels of γδ^+^ and CD8^+^ T cells in lamina propria lymphocytes (LPL) were much higher compared to peripheral blood mononuclear cells. Moreover, the frequency of CD39^+^ CD4^+^ and CD8^+^, but not γδ^+^ LPL positively correlated with T-cell activation. The frequency of CD39^+^ cells among tissue-resident memory LPL (Trm) was higher compared to non-Trm for all subsets, confirming that CD39 is a marker for the tissue-resident memory phenotype. γδ^+^ Trm also showed a distinct cytokine profile upon stimulation – the frequency of IFN-γ^+^ and IL-17A^+^ cells was significantly lower in γδ^+^ Trm compared to non-Trm. Interestingly, we observed a decreased frequency of CD39^+^ γδ^+^ T cells in IBD patients compared to healthy controls (*p* = 0.0049). Prospective studies need to elucidate the exact role of this novel CD39^+^ γδ^+^ T-cell population with tissue-resident memory phenotype and its possible contribution to the pathogenesis of IBD and other inflammatory disorders.

## Introduction

Inflammatory bowel disease (IBD) is the umbrella term for Crohn’s disease (CD), ulcerative colitis (UC) and indeterminate colitis (IC). These diseases share aetiological and pathophysiological features and are characterized by a combination of genetic and environmental factors causing immune dysregulation (1, 2), and altered composition of the gut microbiota (3, 4). As a result, chronic inflammation of the gastrointestinal tract occurs along with a loss of epithelial barrier function (5). Clinically, these diseases are characterized by symptoms like diarrhoea, abdominal pain, fatigue, weight loss, and extraintestinal manifestations (6, 7).

Lymphocytes of the large intestine are exposed to high levels of extracellular adenosine triphosphate (ATP) secreted by i.a. commensal bacteria (8, 9). The balance between extracellular ATP and its metabolite adenosine has been identified as a major factor controlling inflammation in the intestinal milieu (10, 11). On the surface of T cells, ATP can bind to purinergic receptors (e.g., P2X7) leading to an increased Ca^2+^ influx and an enhanced cellular activation (12), whereas adenosine generally dampens the T-cell effector functions by binding to P1 receptors such as the A2A-receptor (11, 13). The degradation of extracellular ATP to adenosine is controlled by the ectoenzymes CD39 and CD73 (14–16). In mouse models of dextrane sulfate sodium (DSS)-induced colitis, genetic deletion of either CD39 (17) or CD73 (18) caused exacerbation of disease. In humans, single nucleotide polymorphisms of the human ENTPD1 gene that lead to a decreased expression of CD39 are associated with increased susceptibility to Crohn’s disease (17). In 2018, Raczkowski et al. demonstrated that CD39^+^ and CD73^+^ cells in human mucosal tissue protect the epithelium from the proinflammatory effects of commensal bacteria-derived ATP in the intestinal lumen (13). Altogether, these findings strongly suggest that CD39 contributes to the regulation of the inflammatory microenvironment, and that changes in CD39 expression or function might promote the onset and perpetuation of IBD. Surprisingly, there are only few human studies that comprehensively assessed the CD39 and CD73 expression patterns of different T-cell subsets in peripheral blood and mucosal tissue of healthy individuals versus IBD patients (19, 20).

Tissue-resident memory cells (Trm) are another T-cell population that has only recently been described, which contributes to the (dys)regulation of the immunological response. These cells are particularly adapted to the intestinal niche (21–23). Specific surface markers for Trm (e.g., CD69 and CD103), as well as distinct transcriptional profiles (24) have been described for these cells. For example, it has been shown that CD69^+^ Trm in the lung are able to initiate potent immune responses via production of IFN-γ and IL-2 while simultaneously displaying a low turnover, thereby preventing excessive inflammation (24). Thus, Trm are of particular interest in the pathogenesis of IBD and display potential new targets for therapeutic approaches (24, 25). Consequently, we wanted to re-evaluate these recent findings in the context of IBD and the contribution of ATP-converting enzymes. In particular, we investigated CD39 and CD73 expression and function of Trm to distinguish them from their recirculating counterparts.

In summary, using several comprehensive 16-color flow cytometry panels, we were able to characterize the peripheral and gut-resident immune cell compositions in healthy individuals and IBD patients. We assessed the expression of CD39 and CD73 together with markers of activation (HLA-DR/CD38), differentiation (CCR7, CD45RA), and tissue-residency (CD69, CD103, CD49a) on CD4^+^ and CD8^+^ T cells and non-conventional subsets like γδ^+^ T cells and mucosa-associated invariant T cells (MAIT) in peripheral blood mononuclear cells (PBMC) and mucosal tissue. Our data hint towards a potential role of CD39^+^ γδ^+^ T cells with tissue-resident memory phenotype in IBD pathogenesis warranting future functional and longitudinal studies focusing on the consequences of their depletion in the mucosa of patients with IBD.

## Materials and Methods

### Study Design

For this study, individuals undergoing colonoscopies were recruited at the University Medical Center Hamburg-Eppendorf. Samples from healthy subjects (*n* = 27) and patients diagnosed with IBD (*n* = 24) were obtained during regular check-up examinations or when patients were referred to the endoscopy unit for further diagnostic exploration. Four to five double biopsies from the colon mucosa were obtained with single-use biopsy forceps and directly processed afterwards. Additionally, we analyzed cryopreserved PBMC from healthy donors (*n* = 9), UC and CD patients (*n* = 10). All individuals gave written, informed consent and this study was approved by the local Institutional Review Board of the Ärztekammer Hamburg (PV5798, PV4444, PV4870) and conducted in accordance with the declaration of Helsinki. Additional information such as clinical symptoms and treatment, co-existing diseases, or the histological analysis of biopsies were extracted from the clinical data bank. Based on the data available, we evaluated the disease status for each patient (26). For an overview of the characteristics of patients who donated gut samples, see Table 1A, for more detailed information about the IBD patients, see Supplementary Tables S1, S2. An overview of the patient characteristics of the analyzed PBMC samples can be found in Table 1B. For a more detailed description, see Supplementary Table S3.

**TABLE 1:** Basic and clinical patient characteristics.

**(A) LPL donors**				
**Characteristics**	**HD**	**CD**	**UC**	**IC**
*n*	30	13	15	1
Female/male	16/14	7/6	9/6	0/1
Median age at sampling (RANGE)	61.3 (34–90)	38.38 (23–58)	45.67 (21–66)	31
**Disease specific medication**				
Mesalazine	-	5	14	1
Azathioprine	-	2	2	1
Corticosteroids	-	1	2	-
Mercaptopurine	-	1	-	
Anti-TNF-α	-	3	1	-
Anti-IL-12 + Anti-IL-23	-	2	-	-
Anti-α4β7	-	1	1	-

**(B) PBMC donors**				

**Characteristics**	**HD**	**CD**	**UC**

*n*	9	5	5
Female/male	5/4	2/3	1/4
Median age at sampling (RANGE)	25,33 (23-29)	48 (28-69)	38,4 (27-54)
**Disease specific medication**			
Mesalazine	-	-	2
Azathioprine	-	1	1
Corticosteroids	-	2	3
Anti-α4β7	-	3	1
Anti-TNF-α	-	1	-

*HD, healthy donors; CD, Crohn‘s disease; UC, ulcerative colitis; IC, indeterminate colitis; LPL, lamina propria lymphocytes; PBMC, peripheral blood mononuclear cells.*

### Sample Acquisition and Processing

Collected in sterile PBS, the samples were processed as previously described (27, 28). In brief, after incubation in Hank’s Balanced Salt Solution (HBSS) containing DTT and EDTA for a short digestion period, they were stored in 6-well, low-binding plates overnight in RPMI supplemented with 10 % FCS, antibiotics and antifungals (1 mg/mL Piperacillin/Tazobactam and 1.25 μg/mL Amphotericin B). The next day, the remaining tissue was disrupted by pipetting and filtered through a 100 μm nylon mesh. After that, the isolated mononuclear cells from the lamina propria were stained and measured immediately. In some cases, the isolated lamina propria lymphocytes (LPL) were cryopreserved and stained later due to the organizational set-up. Frozen PBMC were thawed and stained directly.

### Immune Phenotypic Analysis of Surface and Intracellular Markers of Different Lymphocyte Subsets

Cells were stained with Zombie NIR Fixable Viability stain (BioLegend) and fluorochrome-conjugated antibodies (Supplementary Table S4).

Certain samples were stained intracellularly as well using the FOXP3 Fix/Perm buffer set (eBiosciences, San Diego, CA, United States) (29) according to the manufacturer’s protocol. The samples were stained with the following fluorochrome-labeled antibodies: anti-FOXP3 (AF647, clone: PCH101, eBiosciences, San Diego, CA, United States), anti-IL17A (BV605, clone: BL168), anti-IFN-γ (PE/Dazzle 594, clone: Mab11), anti-IL-10 (BV421, clone: JES3-907), anti-CD4 (PerCP-Cy5.5, clone: SK3) (all Biolegend, London, United Kingdom). For compensation of the panels, single-stained CompBeads (Anti-Mouse Ig,κ/Negative Control Compensation Particles Set, BD Biosciences) were used. As a surrogate for the dye used for the live/dead staining, we applied the APC-Cy7 conjugated anti-CD14 antibody (Biolegend, London, United Kingdom). All samples were analyzed on a BD LSR Fortessa flow cytometer with FACS Diva version 8 (BD Biosciences) on a PC.

### *In vitro* Stimulation

Before intracellular cytokine stainings (ICS), LPL or PBMC were stimulated with 50 ng/mL PMA and 500 ng/mL Ionomycin (Sigma-Aldrich, Seelze, Germany) and incubated at 37°C and 5% CO_2_ for 5 h. For Panel B, which did not include the measurement of IL-10, we resuspended the cells in RPMI and added Brefeldin A (1 mg/mL, Sigma−Aldrich, Seelze, Germany) after 1 h. For detection of IL-10 (Panel C), we resuspended the cells in X-Vivo Medium (Lonza Walkersville Inc., United States) and after 1 h, we added Brefeldin A and Monensin (2 mM, BioLegend, London, United Kingdom). After 5 h, the cells were washed with 2 mL PBS and stained for flow cytometry. For a detailed portrayal over used LPL samples and conducted experiments, see Supplementary Table S5.

### Data Analysis and Statistics

Cytometric data were analyzed using FlowJo v10.6.2 for Windows (FlowJo, BD, Franklin Lakes, NJ, United States). For statistical analysis, GraphPad Prism version 7.01 for Windows (GraphPad Software, Inc., La Jolla, CA, United States) was used. For multiple comparisons we computed two-way ANOVAs, whereas for single comparisons we used Mann–Whitney U tests. For matched analysis, we performed Wilcoxon matched-pairs signed rank tests. Before correlation analysis, we tested the expression of the markers analyzed for Gaussian distribution. If d’Agostino and Pearson normality test were passed, we applied Pearson’s correlation and coefficient for bivariate correlation analysis. If not, Spearman correlation was implemented. In the text, we describe frequencies as means unless stated otherwise. The data on the graphs are expressed as means +/- standard deviation. A *p*-value equal or less than 0.05 was considered significant. *p*-Values are displayed as follows: *p < 0.05, **p < 0.01, ***p < 0.001, ****p < 0.0001. Not significant: ns; *p* > 0.05. For the t-distributed Stochastic Neighbor Embedding (t-SNE) analysis, we used the t-SNE plugin in Flowjo version 10.6.2. Downsampling to 15,000 events was performed on seven healthy donors followed by followed by concatenation into one file for t-SNE analysis (30).

## Results

### LPL and PBMC Differ in Their Relative T-Cell Subset Composition as Well as in Their Expression Patterns of CD39 and CD73

**FIGURE 1:**
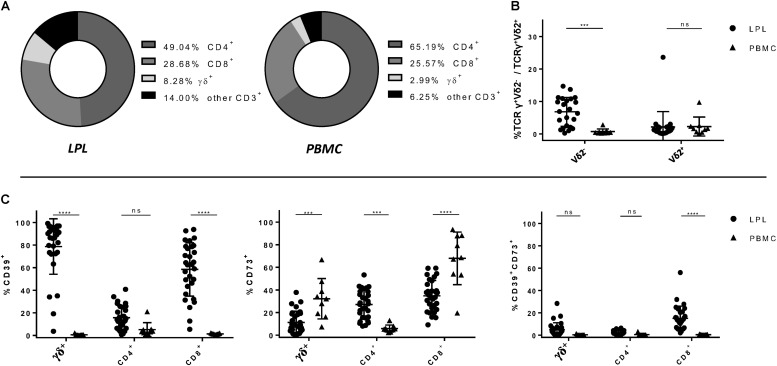
Relative composition of T-cell subsets and frequency of CD39^+^, CD73^+^ and CD39^+^ CD73^+^ T-cell subsets in LPL versus PBMC. **(A)** Frequencies of CD4^+^, CD8^+^ and γδ^+^ T cells isolated from lamina propria (left) and from peripheral blood (right). **(B)** Differences of the frequencies of Vδ2^–^ and Vδ2^+^ T cells between PBMC (round shapes) and LPL (triangular shapes). **(C)** Differences of the frequencies of CD39^+^, CD73^+^ and CD39^+^ CD73^+^ CD4^+^, CD8^+^, and γδ^+^ T cells between LPL and PBMC. Data from healthy donor’s LPL (*n* = 30/23) and PBMC (*n* = 9), presented as means +/- standard deviation. ns ≥ 0.05, *p < 0.05, **p < 0.01, ***p < 0.001, ****p < 0.0001, as calculated by two-way ANOVA. LPL, lamina propria lymphocytes; PBMC, peripheral blood mononuclear cells.

In a first step, we compared peripheral blood with intestinal biopsies from healthy individuals undergoing check-up colonoscopies with respect to the composition of T-cell subsets and their expression of CD39 and CD73. Flow cytometry panels were designed to differentiate between CD4^+^, CD8^+^, MAIT, and γδ^+^ T cells which were further separated into Vδ2^–^ and Vδ2^+^ subsets for some analysis. The gating strategy is shown in Supplementary Figure S1. In line with other reports (31, 32), CD4^+^ T cells were the most frequent T-cell population in both peripheral blood and the lamina propria of the gut epithelium. However, while CD4^+^ T cells were more frequent in PBMC compared to LPL, the proportion of CD8^+^ and γδ^+^ T cells was higher in LPL than in PBMC (Figure 1A). Moreover, we observed an accumulation of Vδ2^–^ γδ^+^ T cells in the gut mucosa compared to peripheral blood of healthy individuals (LPL 7.05% vs PBMC 0.72%, *p* = 0.0005) (Figure 1B). Analysis of CD39 and CD73 expression showed that the frequency of CD39^+^ cells was significantly higher in gut-resident γδ^+^ and CD8^+^ T cells compared to the peripheral blood (γδ^+^: LPL 81.23% vs PBMC 0.577%, *p* < 0.0001; CD8^+^: LPL 59,37% vs PBMC 1.322%, *p* < 0.0001), while there was no significant difference between peripheral and gut-resident CD4^+^ T cells (Figure 1C). By contrast, the frequency of CD73^+^ cells was significantly lower on CD8^+^ and γδ^+^ LPL, but was increased on CD4^+^ LPL (Figure 1C). The frequency of CD39^+^CD73^+^ cells was increased among gut-derived CD8^+^ T cells in comparison to peripheral CD8^+^ T cells. Altogether, in healthy individuals the frequency of CD39^+^ and CD73^+^ T cells considerably differed between PBMC and LPL for the respective subsets.

### LPL of IBD Patients Show Reduced Frequencies of CD39^+^ γδ^+^ T Cells

**FIGURE 2:**
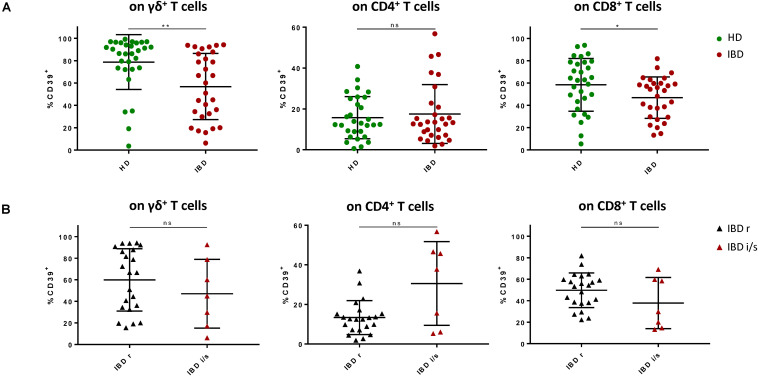
Frequency of CD39^+^ LPL is decreased among γδ^+^ and CD8^+^ T cells of patients with IBD compared to healthy donors. **(A)** Frequencies of CD39^+^ γδ^+^, CD8^+^, and CD4^+^ T cells from healthy donors (green, round shapes) and patients with IBD (red, round shapes). **(B)** Frequencies of CD39^+^ γδ^+^, CD8^+^, and CD4^+^ T cells from IBD patients in remission (black, triangular shapes) and patients with intermediate or severe disease activity (red, triangular shapes). Data from LPL of healthy donors (*n* = 30) and IBD patients (*n* = 29), presented as means +/- standard deviation. ns ≥ 0.05, *p < 0.05, **p < 0.01, ***p < 0.001, ****p < 0.0001, as calculated by Mann–Whitney U test. LPL, lamina propria lymphocytes; HD, healthy donor; IBD, patient with inflammatory bowel disease; IBD r, IBD patient in remission; IBD i/s, IBD patient with intermediate or severe disease activity.

Next, we compared mucosa-derived T cells of IBD patients to those of healthy individuals. We observed no significant differences in the overall frequencies of CD4^+^, CD8^+^, γδ^+^ T cells, and MAIT (Supplementary Figure S2A). However, we observed a decreased frequency of CD39^+^ cells among CD8^+^ and γδ^+^ T cells in individuals diagnosed with IBD compared to healthy donors (γδ^+^: HD 78.36% vs IBD 57.65%, *p* = 0.0049; CD8^+^: HD 59.37% vs IBD 47.45%, *p* = 0.0325) (Figure 2A). This reduction of CD39^+^ CD8^+^ and γδ^+^ T cells did not seem to normalize after treatment as there was no significant difference between LPL from patients in remission and patients with intermediate or severe disease activity (Figure 2B). Gut-derived CD4^+^ T cells did not show significant differences in the CD39 expression level between healthy controls and patients, but we observed a trend towards a higher frequency of CD39^+^ CD4^+^ T cells in patients with intermediate or severe disease activity compared to those patients who were in full remission (Figure 2B). In the MAIT population, there was no difference regarding the frequency of CD39^+^ cells present (Supplementary Figure S2B). Furthermore, we did not observe any differences in the frequencies of CD73^+^ and CD39^+^ CD73^+^ cells among the CD4^+^, CD8^+^, γδ^+^ T cells, and MAIT subsets between the healthy donors and the patient group (Supplementary Figure S3).

### CD39 Expression of Gut-Derived CD4^+^ and CD8^+^, but Not γδ^+^ T Cells, Was Associated With Higher Expression of Activation Markers

**FIGURE 3:**
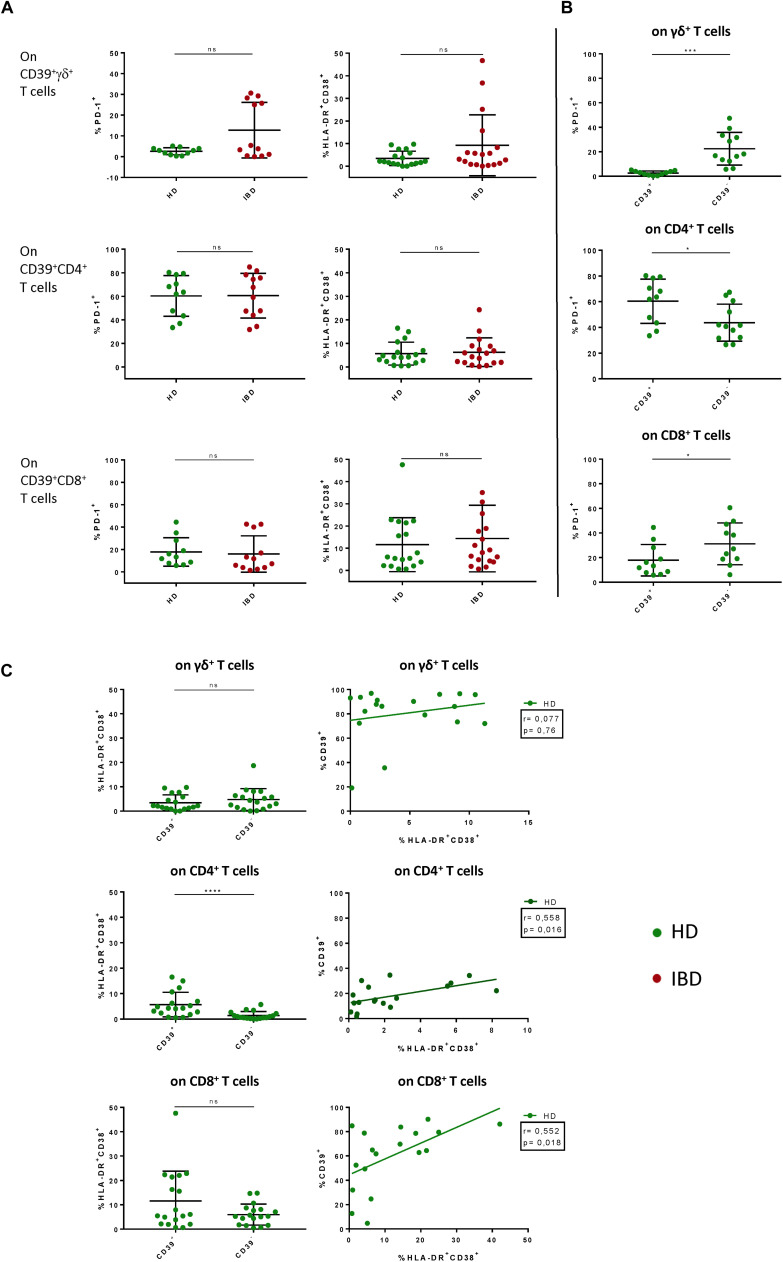
Comparative analysis of PD-1, HLA-DR and CD38 on CD39^+^ γδ^+^, CD4^+^, and CD8^+^ T cells in LPL of HD versus IBD and CD39^+^ versus CD39^–^ LPL in HD. **(A)** Comparison of the frequencies of PD-1^+^ and HLA-DR^+^ CD38^+^ CD39^+^ T cells between healthy donors (green shapes) and patients with IBD (red shapes). **(B)** Comparison of the frequencies of PD-1^+^ and HLA-DR^+^CD38^+^ cells between CD39^+^ and CD39^–^ γδ^+^ T cells of healthy controls. **(C)** Correlation between frequencies of CD39^+^ and HLA-DR^+^CD38^+^ T cells. Spearman correlation analysis was applied. Data from LPL of healthy donors (*n* = 11/18) and IBD patients (*n* = 12/18), presented as means +/- standard deviation. ns ≥ 0,05, *p < 0.05, **p < 0.01, ***p < 0.001, ****p < 0.0001, as calculated by Mann–Whitney U test **(A)**, Wilcoxon matched-pairs signed rank test **(B)** + **(C)**, and Spearman correlation **(C)**. HD, healthy donor; IBD, patient with inflammatory bowel disease; LPL, lamina propria lymphocytes.

Since we only observed differences in CD39 expression between healthy donors and IBD patients we put particular focus on the thorough analysis of CD39^+^ T cells. To assess the activation status of CD39^+^ T cells, we also analyzed the expression of traditional markers associated with T-cell activation and exhaustion (co-expression of HLA-DR and CD38, PD-1). We did not find any significant differences between CD39^+^ T cells from healthy individuals and IBD patients, either for the frequency of PD-1^+^ or HLA-DR/CD38 double-positive T cells (Figure 3A). In contrast, studying CD39^+^ versus CD39^–^ T cells in healthy individuals revealed significant differences: regarding gut-resident γδ^+^ and CD8^+^ T cells, the frequency of PD-1^+^ cells was significantly lower among CD39^+^ compared to CD39^–^ cells (γδ^+^: 2.41% vs 23.25%, *p* = 0.002; CD8^+^: 18.01% vs 32.96%, *p* = 0.0371) (Figure 3B). In contrast, the frequency of PD-1^+^ cells was significantly higher on CD39^+^ compared to CD39^–^ CD4^+^ T cells (59.33% vs 43.9%, *p* = 0.0488) (Figure 3B). However, further analysis did not reveal any correlation between the expression of CD39 and PD-1 for any T-cell subset (data not shown).

With respect to T-cell activation, we observed a significantly higher frequency of HLA-DR/CD38 co-expressing cells among CD39^+^ compared to CD39^–^ CD4^+^ T cells, and also a trend towards a higher frequency in the CD39^+^ CD8^+^ T-cell subset (CD4^+^: 5.839% vs 1.475%, *p* < 0.0001; CD8^+^: 12.19% vs 6.323%, *p* = 0.0797) (Figure 3C). Next, we performed correlation analyses to determine whether CD39 expression was associated with enhanced T-cell activation. Indeed, the frequency of HLA-DR/CD38 double-positive T cells correlated with the frequency of CD39^+^ cells of the CD4^+^ and CD8^+^ T-cell compartment. Interestingly, the γδ^+^ T cells did not show elevated levels of activation markers among CD39^+^ γδ^+^ T cells compared to CD39^–^ γδ^+^ T cells (3.611% vs 4.987%, *p* = 0.1729) (Figure 3C). There were no significant differences detectable for the frequency of HLA-DR^+^/CD38^+^ and PD-1^+^ cells when we compared CD39^+^ and CD39^–^ T-cell subsets from healthy individuals with those from IBD patients (Supplementary Figure S4A). In sum, gut-derived CD39^+^ γδ^+^ T cells in healthy donors were characterized by low expression of HLA-DR, CD38 and PD-1. While preserving this phenotype, the frequency of CD39^+^ γδ^+^ T cells in IBD patients was significantly decreased compared to healthy donors.

### CD39 Expression Is Associated With Different Effector Cytokine Profiles of Peripheral Versus Intestinal γδ^+^ and CD4^+^ T Cells

**FIGURE 4:**
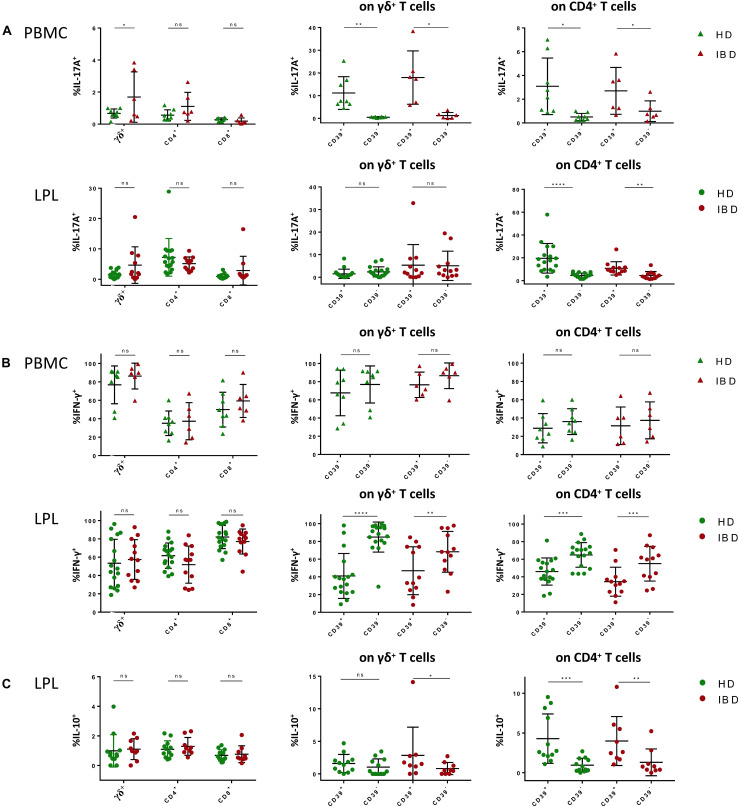
ICS after short term stimulation of γδ^+^, CD4^+^, and CD8^+^ T cells in PBMC and LPL with PMA/Ionomycin. **(A)** (Left) Frequencies of IL-17A^+^ γδ^+^, CD4^+^, and CD8^+^ T cells from healthy donors (green) and patients with IBD (red); (center) Comparison of the frequencies of IL-17A^+^ cells between CD39^+^ and CD39^–^ γδ^+^ T cells; (right) Comparison of the frequencies of IL-17A^+^ cells between CD39^+^ and CD39^–^ CD4^+^ T cells; data from PBMC in upper row (triangular shapes), data from LPL in bottom row (round shapes). **(B)** (Left) Frequencies of IFN-γ^+^ γδ^+^, CD4^+^ and CD8^+^ T cells from healthy donors (green) and patients with IBD (red); (center) Comparison of the frequencies of IFN-γ^+^ cells between CD39^+^ and CD39^–^ γδ^+^ T cells; (right) Comparison of the frequencies of IFN-γ^+^ between CD39^+^ and CD39^–^ CD4^+^ T cells; data from PBMC in upper row (triangular shapes), data from LPL in bottom row (round shapes). **(C)** (Left) Frequencies of IL-10^+^ γδ^+^, CD4^+^ and CD8^+^ T cells from healthy donors (green) and patients with IBD (red); (center) Comparison of the frequencies of IL-10^+^ cells between CD39^+^ and CD39^–^ γδ^+^ T cells; (right) Comparison of the frequencies of IL-10^+^ cells between CD39^+^ and CD39^–^ CD4^+^ T cells; data from LPL (round shapes). Data from healthy donor’s LPL (*n* = 17/12) and PBMC (*n* = 8) and IBD patients’ LPL (*n* = 11/9) and PBMC (*n* = 6), presented as means +/- standard deviation. ns ≥ 0.05, *p < 0.05, **p < 0.01, ***p < 0.001, ****p < 0.0001, as calculated by two-way ANOVA and Wilcoxon matched-pairs signed rank test (comparison of CD39^+^ and CD39^–^ cells). HD, healthy donor; IBD, patient with inflammatory bowel disease; LPL, lamina propria lymphocytes; PBMC, peripheral blood mononuclear cells; ICS, intracellular cytokine staining.

Next, we wanted to gain further insight into the functionality of CD39^+^ LPL and PBMC. Thus, we stimulated blood- and gut-derived lymphocytes and performed ICS to assess the frequencies of IL-17A^+^, IFN-γ^+^, and IL-10^+^ cells (exemplary plots: Supplementary Figure S5). After 5 h of stimulation with PMA/Ionomycin, peripheral γδ^+^ T cells from IBD patients displayed significantly higher frequencies of IL-17A^+^ cells compared to peripheral γδ^+^ T cells from healthy donors (IBD 1.687% vs HD 0.666%, *p* = 0.0326) (Figure 4A), contrary to peripheral CD4^+^ or CD8^+^ T cells which displayed no differences in IL-17A expression (Figure 4A).

Surprisingly, the small population of peripheral CD39^+^ γδ^+^ T cells consisted of potent IL-17A producers with significantly higher frequencies of IL-17A^+^ cells than in the CD39^–^ γδ^+^ T-cell population (healthy: 11.16% vs 0.519%, *p* = 0.0078). Also, CD39^+^ CD4^+^ T cells showed higher frequencies of IL-17A-producing cells compared to CD39^–^ CD4^+^ T cells (Figure 4A). We therefore wondered whether the gut-derived γδ^+^ and CD4^+^ T cells had similar characteristics. In LPL, we did not see differences in IL-17A production between healthy individuals and IBD patients for any of the subsets analyzed. As observed in PBMC, CD39^+^ CD4^+^ LPL displayed a higher frequency of IL-17A^+^ cells than CD39^–^ CD4^+^ LPL. In contrast to PBMC, the comparison of CD39^+^ and CD39^–^ gut-derived γδ^+^ T cells did not show significant differences in the frequencies of IL-17A-producing cells.

We observed no significant difference of the frequency of IFN-γ-producing cells between peripheral CD4^+^, CD8^+^, or γδ^+^ T cells of healthy individuals and the respective subsets from patients suffering from IBD (Figure 4B). Moreover, we did not find any association between CD39 expression and IFN-γ production in the aforementioned peripheral T-cell subsets. In LPL, there were also no differences detectable in terms of IFN-γ production between the patients and the control group for any of the subsets. However, comparing CD39^+^ with their CD39^–^ counterparts, CD39^+^ γδ^+^ T cells as well as CD39^+^ CD4^+^ T cells showed a significantly lower frequency of IFN-γ^+^ cells (healthy: CD4^+^: 47.12% vs 64.57%, *p* = 0.0004; γδ^+^: 43.16% vs 85.61%, *p* = 0.0001) (Figure 4B). To investigate whether CD39^+^ T cells have a rather tolerance inducing role in the gut environment, we next looked for IL-10 production of these cells (33–36). Notably, CD39^+^ CD4^+^ as well as CD39^+^ γδ^+^ T cells of IBD patients showed a higher frequency of IL-10^+^ cells than their CD39^–^ counterparts (CD4^+^: 3.988% vs 1.309%, *p* = 0.0102; γδ^+^: 2.847% vs 0.821%, *p* = 0.023) (Figure 4C). Taken together, CD4^+^ T cells seem to maintain their IL-17^high^IFN-γ^low^ phenotype when migrating from peripheral blood into the gut mucosa. In contrast, CD39^+^ γδ^+^ T cells from peripheral blood display an IL-17A^high^IFN-γ^high^ phenotype which is different from the IL-17A^low^IFN-γ^low^ phenotype displayed by CD39^+^ γδ^+^ LPL. Gut-derived CD39^+^ γδ^+^ T cells were furthermore able to produce IL-10 in samples from healthy donors and patients with IBD.

### The Majority of CD8^+^ and γδ^+^ LPL Display a Tissue-Resident Memory Phenotype That Is Associated With High Expression of CD39

**FIGURE 5:**
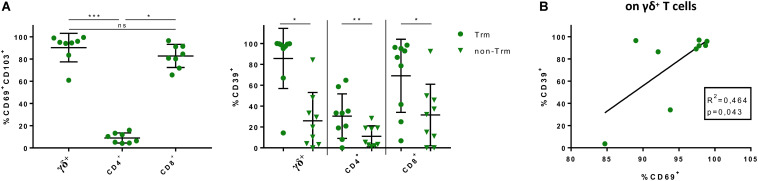
Frequencies of CD69^+^ CD103^+^ γδ^+^, CD4^+^ and CD8^+^ LPL; CD39 as possible signature marker for Trm. **(A)** (Left) Frequencies of CD69^+^ CD103^+^ γδ^+^, CD4^+^, and CD8^+^ LPL from healthy donors, two data points were removed after testing for outliers; (right) Comparison of CD39^+^ γδ^+^, CD4^+^, and CD8^+^ Trm (round shapes) and non-Trm (triangular shapes) from healthy donors. **(B)** Correlation between frequency of CD69^+^ and CD39^+^ γδ^+^ LPL from healthy donors. *R*^2^ denotes Pearson’s coefficient and p-value. Data of LPL of healthy donors (*n* = 8/9), presented as means +/- standard deviation. ns ≥ 0.05, *p < 0.05, **p < 0.01, ***p < 0.001, ****p < 0.0001, as calculated by Friedman test with Dunn’s multiple comparisons test (**A**, left graph), Wilcoxon matched-pairs signed rank test (**A**, right graph) and Pearson’s correlation analysis **(B)**. Trm, tissue-resident memory cells; LPL, lamina propria lymphocytes.

Taking the major differences in CD39 expression between blood and gut and the unique cytokine profile of gut-resident CD39^+^ γδ^+^ T cells into account, we asked how the aforementioned CD39^+^ T-cell populations might actually represent Trm. These Trm were effector memory cells that we identified via CD69 and CD103 expression (for gating strategy, see Supplementary Figure S1B). As described before by Mackay et al. (37), we found significantly higher frequencies of CD69^+^CD103^+^ Trm among CD8^+^ and γδ^+^ T cells than among CD4^+^ T cells in the intestinal lamina propria of healthy individuals (Figure 5A). We compared the frequency of CD39^+^ cells among CD69^+^CD103^+^ Trm with the non-Trm population (cells expressing none or only one of the markers, Supplementary Figure S1B). The frequency of CD39^+^ cells was significantly higher among Trm in all subsets (γδ^+^: 85.7% vs 25.86%, *p* = 0.0117; CD4^+^: 30.37% vs 11.05%, *p* = 0.0078; CD8^+^: 69.06% vs 31.52%, *p* = 0.0117) (Figure 5A).

### CD39 as Trm Marker of γδ^+^ T Cells

**FIGURE 6:**
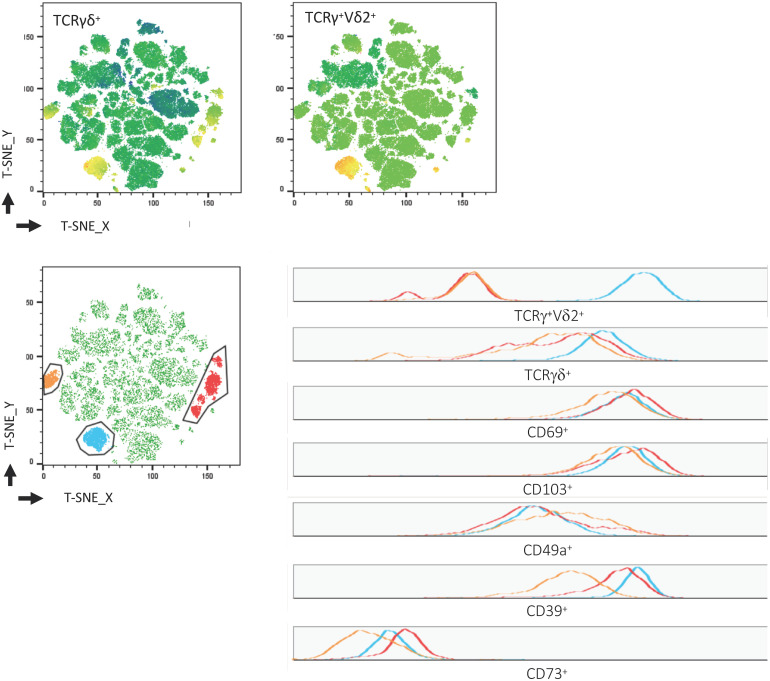
Visualization of γδ^+^ Trm among CD3^+^ LPL. T-SNE map created from a concatenated file of CD3^+^ LPL of healthy donors (*n* = 7). Clusters of TCRγδ^+^ and TCRγ^+^Vδ2^+^ cells **(top)**. TCRγ^+^Vδ2^+^ (blue) and TCRγ^+^Vδ2^–^ (red and orange) were manually gated and overlaid on total CD3^+^ cells **(bottom, left)**. Expression of TCRγδ^+^, TCRγ^+^Vδ2^+^, CD69, CD103, CD49a, CD39, CD73 within the gated populations is depicted in histograms **(bottom, right)**. Trm, tissue-resident memory cells; LPL, lamina propria lymphocytes.

In addition to the elevated frequency of CD39^+^ cells within Trm, we found a positive correlation between the well-established tissue residency marker CD69 (24, 38) and CD39 on γδ^+^ T cells (*p* = 0.043, *R*^2^ = 0.464) (Figure 5B). To confirm our hypothesis of the existence of this tissue-resident γδ^+^ T-cell population, we performed a t-SNE analysis. As shown in Figure 6, three clusters representing γδ^+^ T cells could be readily identified based on their expression of the γδ T-cell receptor. One cluster corresponded to Vδ2^+^ γδ^+^ T cells while Vδ2^–^ γδ^+^ T cells were divided into two clusters. The distribution of CD8^+^ and CD4^+^ T cells was more heterogeneous (data not shown). All three clusters of γδ^+^ T cells expressed CD39, CD69, CD103, and CD49a, while they were negative for CD73.

**FIGURE 7:**
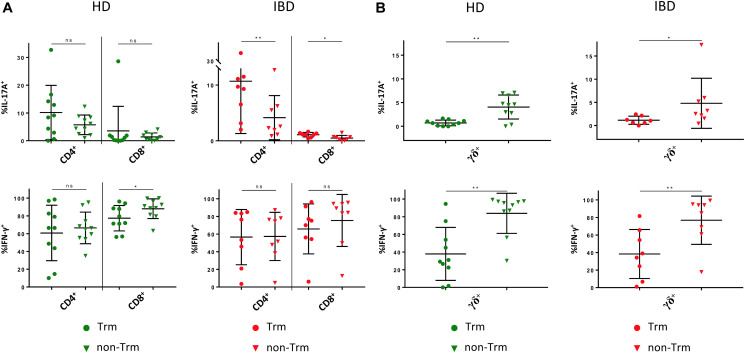
Cytokine production by tissue-resident versus non-tissue-resident CD4^+^, CD8^+^, and γδ^+^ LPL. **(A)** Production of IL-17A and IFN-γ by Trm (round shapes) versus non-Trm (triangular shapes) CD4^+^ and CD8^+^ LPL from healthy donors (green) and IBD patients (red). **(B)** Production of IL-17A and IFN-γ by Trm (round shapes) versus non-Trm (triangular shapes) γδ^+^ LPL from healthy donors (green) and IBD patients (red). Data from LPL of healthy donors (*n* = 10) and IBD patients (*n* = 8), presented as means +/- standard deviation. ns ≥ 0.05, *p < 0.05, **p < 0.01, ***p < 0.001, ****p < 0.0001, as calculated by Wilcoxon matched-pairs signed rank test. LPL, lamina propria lymphocytes; HD, healthy donor; IBD, patient with inflammatory bowel disease; Trm, tissue-resident memory cells.

Next, we evaluated whether the frequency of Trm and their CD39 expression in LPL of IBD patients was different from healthy donors. Interestingly, we were not able to detect significant differences regarding the frequency of Trm and CD39^+^ Trm either for CD4^+^, CD8^+^, or γδ^+^ T cells (Supplementary Figure S4B). However, we observed a trend towards a lower frequency of γδ^+^ Trm and CD39^+^ γδ^+^ Trm in IBD patients compared to healthy donors (γδ^+^ Trm: HD 84.5% vs IBD 67.64%; *p* = 0.2089; CD39^+^ γδ^+^ Trm: HD 83.94% vs IBD 73.21%; *p* = 0.7955) (Supplementary Figure S4B). We then compared cytokine production between Trm and non-Trm in patients and healthy controls. We found higher frequencies of IL-17A^+^ cells among the CD4^+^ Trm compared to the CD4^+^ non-Trm subset. This difference became significant in samples from IBD patients (Figure 7A). CD8^+^ Trm also showed significantly higher frequencies of IL-17A^+^ cells compared to CD8^+^ non-Trm in the context of IBD. This difference was not apparent in healthy donors. We did not observe significant differences in IFN-γ production between CD4^+^ Trm and non-Trm either in controls or patients. The frequency of IFN-γ^+^ CD8^+^ Trm from lamina propria of healthy controls was significantly lower compared to non-Trm. Remarkably, γδ^+^ Trm displayed significantly lower frequencies of IL-17A^+^ and IFN-γ^+^ cells compared to their non-Trm counterparts in both healthy donors and IBD patients (healthy: γδ^+^ IL17A^+^: 0.709% vs 4.051%, *p* = 0.0078; γδ^+^ IFN-γ^+^: 37.95% vs 83.85%, *p* = 0.002; IBD: γδ^+^ IL17A^+^: 1.14% vs 4.799%, *p* = 0.0469; γδ^+^ IFN-γ^+^: 38.37% vs 76.83%, *p* = 0.0078) (Figure 7B). Taken together, the IL-17A^low^IFN-γ^low^ phenotype of CD39^+^ γδ^+^ T cells is in line with the IL-17A^low^IFN-γ^low^ phenotype of γδ^+^ Trm. Altogether, these results support the notion that CD39 can be used as Trm marker - especially for γδ^+^ T cells.

## Discussion

It is commonly agreed that the immune response in the mucosal compartment is profoundly shaped by extracellular signaling of ATP and adenosine (10, 11, 39). Several gut-derived cell populations can modulate their CD39 expression and thereby influence ATP/adenosine levels (36, 40, 41). We found a highly significant difference of the CD39 and CD73 expression of CD4^+^, CD8^+^ and γδ^+^ T cells between LPL and PBMC highlighting the peculiarity of the mucosal compartment where T cells adapt their phenotype and their effector functions to this special environment (21, 22, 42). In particular, peripheral CD4^+^, CD8^+^ and γδ^+^ T cells displayed a CD39^low^CD73^high^ phenotype compared to gut-derived CD8^+^ and γδ^+^ T cells which showed a CD39^high^CD73^low^ phenotype.

Further details about the regulation of CD39 expression on T cells in humans need to be understood: The change of phenotype and increased expression of CD39 in the gut suggests a general association with T-cell activation (13), but increased hypoxia levels in the mucosal tissue also seem to be involved (43, 44). Hypoxia leads to elevated levels of the transcription factors HIF-1α and Sp1 which downstream lead to the upregulation of CD39 surface expression (45).

Surprisingly, we only identified small differences in the expression patterns of CD39 and CD73 between healthy controls and IBD patients. It will be important to study whether greater differences in terms of frequency, phenotype or function will be evident when analyzing larger cohorts of untreated patients with more severe IBD disease activity. Additionally, it would be interesting to distinguish between treatment groups and patients with UC or CD.

A key finding of our study was the decreased frequency of gut-derived CD39^+^ γδ^+^ T cells in IBD patients regardless of disease severity compared to healthy donors. Since most of the gut-resident γδ^+^ LPL did not express the Vδ2 chain of the γδ T-cell receptor, we postulate that our observations are applicable to Vδ1^+^ γδ^+^ T cells as they are the dominant γδ^+^ T-cell subset in the gut (46). We assume that the reduced frequency of CD39^+^ γδ^+^ T cells could indeed be crucial for the development of IBD. In a murine model of DSS-induced colitis, it was previously shown that elevated, extracellular ATP levels are associated with progression of disease (47). Consistent with a regulatory function of CD39^+^ cells (34, 36, 48, 49), Otsuka et al. recently described a distinct subset of γδ^+^ T cells in mice that was characterized by CD39 expression and sufficiently suppressed proliferation of and cytokine production by effector T cells (33). Thus, enzymatic activity and degradation of ATP of CD39^+^ γδ^+^ T cells might promote an inhibitory environment and should be monitored in future experiments.

Regulatory T cells (Tregs, CD4^+^ CD25^+^ FOXP3^+^) have been described to play an important role suppressing inflammation in IBD (50–52). However, there are contradicting reports about their relative frequency in the gut (52–55) and only little is known about their interaction with γδ^+^ T cells (56, 57). In a small subset of patients, we analyzed gut derived Tregs alongside with γδ^+^ T cells. Neither the frequencies of Tregs in tissue of IBD patients compared to healthy donors nor CD39 expression significantly differed in this small sample size (Supplementary Figure S6A). However, when we performed a correlation analysis to see how Treg and CD39^+^ γδ^+^ T cell frequencies would correspond to each other, we found a negative association in IBD patients but not in healthy controls (Supplementary Figure S6B). This could indicate that an increase of Tregs in the mucosal department might be a compensatory mechanism to counteract the decrease of CD39^+^ γδ^+^ T cells. To better integrate this observation in the immune landscape of regulatory T cell subsets and to validate the overall frequencies of Tregs and their CD39 expression in the gut, prospective, more detailed studies with larger cohorts need to be performed.

Importantly, in IBD patients CD39^+^ γδ^+^ LPL displayed only low *ex vivo* FOXP3 expression but the frequency of FOXP3^+^ CD39^+^ γδ^+^ LPL was significantly higher compared to healthy controls (*p* = 0.0221), (Supplementary Figure S6C). In future follow up studies it will be interesting to elucidate the detailed molecular signature of CD39^+^ γδ^+^ LPL with respect to the expression of other regulatory molecules and transcription factors.

**FIGURE 8:**
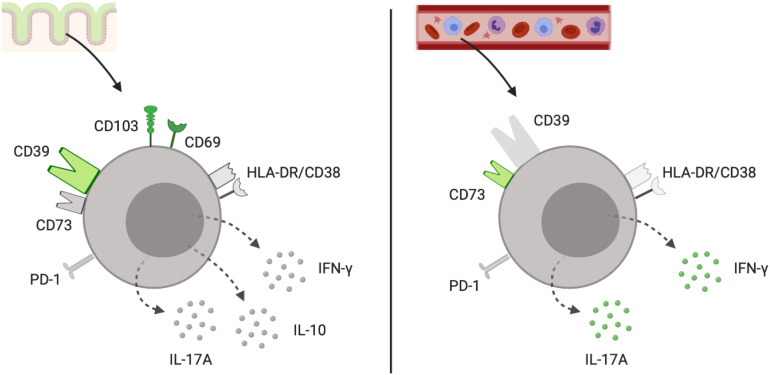
Schematic representation of the phenotype and functional profile of tissue-resident CD39^+^ γδ^+^ LPL and peripheral CD39^+^ γδ^+^ T cells. Surface markers and intracellular cytokines colored in grey (low frequency) and green (high frequency) for CD39^+^ γδ^+^ Trm of the large intestine **(left)** and CD39^+^ γδ^+^ T cells from peripheral blood **(right)**. Illustration created with the online software BioRender (San Francisco, CA, United States). Trm, tissue-resident memory cells.

In our *in vitro* experiments, the CD39^+^ γδ^+^ T cells exhibited an IL-17A^low^IFN-γ^low^ phenotype in contrast to the other CD39^+^ T-cell subpopulations in the gut. Furthermore, we found that CD39^+^ γδ^+^ T cells were able to produce IL-10 upon stimulation. The frequencies of IL-10^+^ CD39^+^ γδ^+^ were lower than of IL-10^+^ CD39^+^ CD4^+^ T cells but significantly higher compared to their IL-10^+^ CD39^–^ γδ^+^ counterparts. The main findings regarding phenotypic and functional properties of CD39^+^ γδ^+^ LPL in contrast to peripheral CD39^+^ γδ^+^ are summarized in Figure 8. One hypothesis that needs to be further explored is whether the loss of CD39^+^ γδ^+^ T cells that are able to produce IL-10 and upregulate regulatory transcriptional factors like FOXP3 in the gut mucosa of IBD patients plays an important role in the onset and perpetuation of IBD.

Lymphocytes in the gut mucosa are constantly exposed to a special environment that features both commensal bacteria and potential pathogens (58). Hence, a newly defined Trm population for first-line defense is present which we hypothesize is playing a key role in modulating the immune homeostasis of the large intestine. The important Trm marker CD69 is commonly used as an early activation marker (59), but it was shown that CD69 expression is not associated with recent activation in mucosal tissue (24). Of note, CD69 expressed on LPL interacts with the Sphingosine-1-phosphate-receptor-1 and therefore prevents tissue egress (60, 61). Synergistically, the integrin CD103 binds to E-cadherin which is highly expressed on epithelial cells (62). As a consequence, the expression of CD69 and CD103 prevents T cells from recirculation between tissue and blood and their surface expression can be used to identify Trm in the intestine (23, 63). In line with Zundler et al. who described increased frequencies of CD4^+^ Trm in LPL of IBD patients compared to healthy donors (64), we observed an accumulation of CD4^+^ Trm in the mucosa from patients with IBD. Moreover, we detected a slightly decreased frequency of γδ^+^ Trm in IBD. We furthermore established a link between CD39 expression and γδ^+^ Trm, indicating the decreased frequency of CD39^+^ γδ^+^ LPL in IBD mirrors the loss of γδ^+^ Trm. So far, this loss has been shown for intraepithelial CD39^+^ γδ^+^ and CD8^+^ lymphocytes (19, 20). Our results confirm and extend these findings to the lamina propria of the large intestine in healthy individuals and patients with IBD.

Other studies postulate that rather than immunosuppressive activity, the interaction of Trm with dendritic cells is crucial for protection of the epithelial tissue in IBD (19). In contrast, data of Trm promoting inflammation have been published (64, 65). To our knowledge, our data are the first indicating that CD39^+^ γδ^+^ Trm might play a central, tolerance modulating role in the gut mucosa since significantly lower frequencies of Trm produce IFN-γ and IL-17A compared to non-Trm. Their impaired frequency in IBD patients strongly suggests that they are involved in the pathogenesis of this disease. Future studies should focus on investigating the direct immunosuppressive activity of CD39^+^ γδ^+^ Trm by *in vitro* inhibition assays.

However, several limitations of the current study should be noted that are inherent to heterogeneous, cross-sectionally investigated cohorts of patients with CD, UC, and IC. Patients were under different disease-specific treatments and some suffered from concomitant diseases like primary sclerosing cholangitis or autoimmune hepatitis. Additionally, three patients had a transplanted liver and received further immunosuppressive treatment. However, further analysis of the transplanted versus not-transplanted IBD patients in this cohort did not reveal significant differences (data not shown). Future studies should be of a prospective design with longitudinal analysis of patients before and under therapy with matched blood and gut samples. Furthermore, it would be interesting how our findings are affected by CD39-encoding ENTPD1 polymorphisms (17).

Our data also seem to support the investigation of future therapeutic approaches that aim to alter purinergic signaling cascades in IBD patients in order to dampen the overall inflammation (66). Treatment suggestions based on murine models include apyrase substitution (47), increase of the extracellular adenosine concentration via mucosa-specific inhibition of adenosine uptake (67), or HIF-1α stabilization (43, 68, 69). The role of CD39-expressing, tissue-resident γδ^+^ LPL should be highlighted and considered for prospective investigations in the treatment of IBD.

In summary, our data give a first comprehensive portrayal of CD39 and CD73 expression patterns on different T-cell populations with and without tissue-resident memory phenotype in the large intestine and peripheral blood of healthy individuals and in the context of IBD.

## Data Availability Statement

All datasets presented in this study are included in the article/Supplementary Material.

## Ethics Statement

The studies involving human participants were reviewed and approved by Institutional Review Board of the Ärztekammer Hamburg. The patients/participants provided their written informed consent to participate in this study.

## Author Contributions

JS and JL designed the study and wrote the first draft of manuscript. JS gave funding. JL, MW, and RW conducted the experiments. MK, DR, JH, and JS obtained biopsies from patients undergoing colonoscopy. JL analyzed the data. JME and MW contributed to the interpretation of the multicolour flow cytometry panels. JL prepared the figures and got input from JS, MW, and all other authors. All authors reviewed the manuscript and gave important input.

## Conflict of Interest

The authors declare that the research was conducted in the absence of any commercial or financial relationships that could be construed as a potential conflict of interest.
