# Supplementary material for: Decreased Frequency of Intestinal CD39+ γδ+ T Cells With Tissue-Resident Memory Phenotype in Inflammatory Bowel Disease
Source: Front Immunol. 2020 Sep 24;11:567472. doi: 10.3389/fimmu.2020.567472 (PMC7541837; doi:10.3389/fimmu.2020.567472)
Supplement: Supplementary file 1 [file Presentation_1.PPTX]

## Slide 1
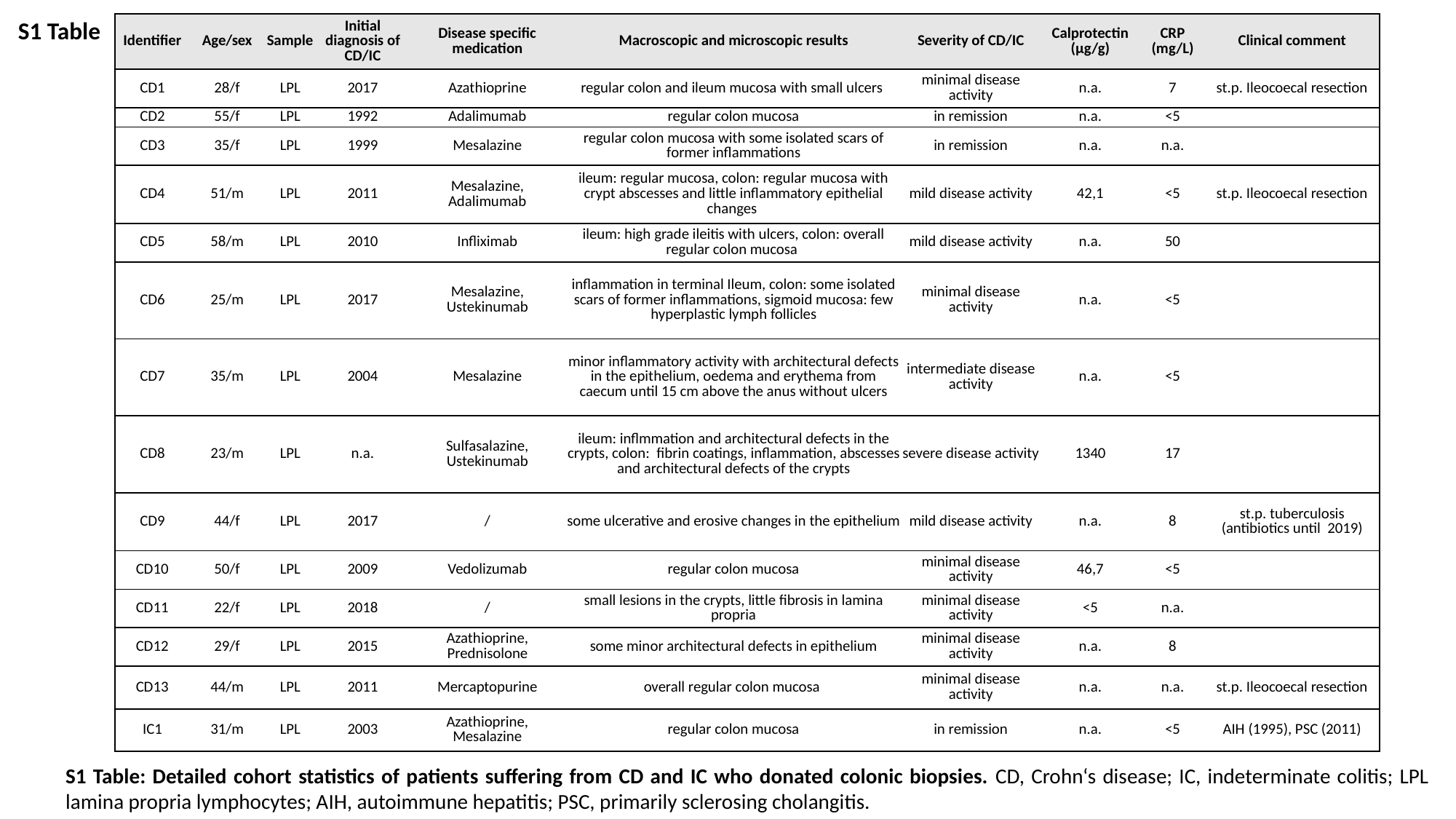

S1 Table
| Identifier | Age/sex | Sample | Initial diagnosis of CD/IC | Disease specific medication | Macroscopic and microscopic results | Severity of CD/IC | Calprotectin (µg/g) | CRP (mg/L) | Clinical comment |
| --- | --- | --- | --- | --- | --- | --- | --- | --- | --- |
| CD1 | 28/f | LPL | 2017 | Azathioprine | regular colon and ileum mucosa with small ulcers | minimal disease activity | n.a. | 7 | st.p. Ileocoecal resection |
| CD2 | 55/f | LPL | 1992 | Adalimumab | regular colon mucosa | in remission | n.a. | <5 | |
| CD3 | 35/f | LPL | 1999 | Mesalazine | regular colon mucosa with some isolated scars of former inflammations | in remission | n.a. | n.a. | |
| CD4 | 51/m | LPL | 2011 | Mesalazine, Adalimumab | ileum: regular mucosa, colon: regular mucosa with crypt abscesses and little inflammatory epithelial changes | mild disease activity | 42,1 | <5 | st.p. Ileocoecal resection |
| CD5 | 58/m | LPL | 2010 | Infliximab | ileum: high grade ileitis with ulcers, colon: overall regular colon mucosa | mild disease activity | n.a. | 50 | |
| CD6 | 25/m | LPL | 2017 | Mesalazine, Ustekinumab | inflammation in terminal Ileum, colon: some isolated scars of former inflammations, sigmoid mucosa: few hyperplastic lymph follicles | minimal disease activity | n.a. | <5 | |
| CD7 | 35/m | LPL | 2004 | Mesalazine | minor inflammatory activity with architectural defects in the epithelium, oedema and erythema from caecum until 15 cm above the anus without ulcers | intermediate disease activity | n.a. | <5 | |
| CD8 | 23/m | LPL | n.a. | Sulfasalazine, Ustekinumab | ileum: inflmmation and architectural defects in the crypts, colon: fibrin coatings, inflammation, abscesses and architectural defects of the crypts | severe disease activity | 1340 | 17 | |
| CD9 | 44/f | LPL | 2017 | / | some ulcerative and erosive changes in the epithelium | mild disease activity | n.a. | 8 | st.p. tuberculosis (antibiotics until 2019) |
| CD10 | 50/f | LPL | 2009 | Vedolizumab | regular colon mucosa | minimal disease activity | 46,7 | <5 | |
| CD11 | 22/f | LPL | 2018 | / | small lesions in the crypts, little fibrosis in lamina propria | minimal disease activity | <5 | n.a. | |
| CD12 | 29/f | LPL | 2015 | Azathioprine, Prednisolone | some minor architectural defects in epithelium | minimal disease activity | n.a. | 8 | |
| CD13 | 44/m | LPL | 2011 | Mercaptopurine | overall regular colon mucosa | minimal disease activity | n.a. | n.a. | st.p. Ileocoecal resection |
| IC1 | 31/m | LPL | 2003 | Azathioprine, Mesalazine | regular colon mucosa | in remission | n.a. | <5 | AIH (1995), PSC (2011) |
S1 Table: Detailed cohort statistics of patients suffering from CD and IC who donated colonic biopsies. CD, Crohn‘s disease; IC, indeterminate colitis; LPL lamina propria lymphocytes; AIH, autoimmune hepatitis; PSC, primarily sclerosing cholangitis.

## Slide 2
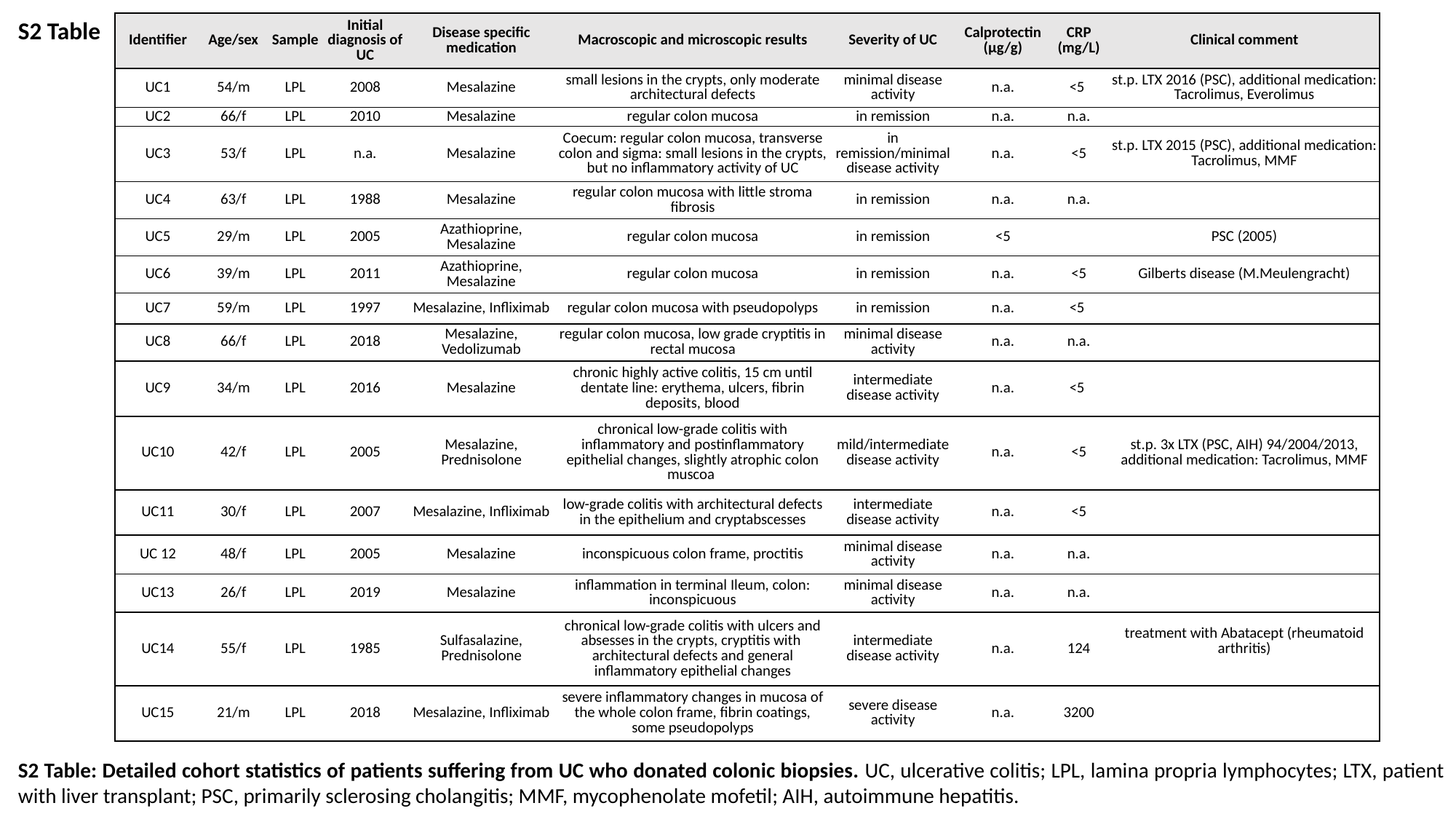

S2 Table
| Identifier | Age/sex | Sample | Initial diagnosis of UC | Disease specific medication | Macroscopic and microscopic results | Severity of UC | Calprotectin (µg/g) | CRP (mg/L) | Clinical comment |
| --- | --- | --- | --- | --- | --- | --- | --- | --- | --- |
| UC1 | 54/m | LPL | 2008 | Mesalazine | small lesions in the crypts, only moderate architectural defects | minimal disease activity | n.a. | <5 | st.p. LTX 2016 (PSC), additional medication: Tacrolimus, Everolimus |
| UC2 | 66/f | LPL | 2010 | Mesalazine | regular colon mucosa | in remission | n.a. | n.a. | |
| UC3 | 53/f | LPL | n.a. | Mesalazine | Coecum: regular colon mucosa, transverse colon and sigma: small lesions in the crypts, but no inflammatory activity of UC | in remission/minimal disease activity | n.a. | <5 | st.p. LTX 2015 (PSC), additional medication: Tacrolimus, MMF |
| UC4 | 63/f | LPL | 1988 | Mesalazine | regular colon mucosa with little stroma fibrosis | in remission | n.a. | n.a. | |
| UC5 | 29/m | LPL | 2005 | Azathioprine, Mesalazine | regular colon mucosa | in remission | <5 | | PSC (2005) |
| UC6 | 39/m | LPL | 2011 | Azathioprine, Mesalazine | regular colon mucosa | in remission | n.a. | <5 | Gilberts disease (M.Meulengracht) |
| UC7 | 59/m | LPL | 1997 | Mesalazine, Infliximab | regular colon mucosa with pseudopolyps | in remission | n.a. | <5 | |
| UC8 | 66/f | LPL | 2018 | Mesalazine, Vedolizumab | regular colon mucosa, low grade cryptitis in rectal mucosa | minimal disease activity | n.a. | n.a. | |
| UC9 | 34/m | LPL | 2016 | Mesalazine | chronic highly active colitis, 15 cm until dentate line: erythema, ulcers, fibrin deposits, blood | intermediate disease activity | n.a. | <5 | |
| UC10 | 42/f | LPL | 2005 | Mesalazine, Prednisolone | chronical low-grade colitis with inflammatory and postinflammatory epithelial changes, slightly atrophic colon muscoa | mild/intermediate disease activity | n.a. | <5 | st.p. 3x LTX (PSC, AIH) 94/2004/2013, additional medication: Tacrolimus, MMF |
| UC11 | 30/f | LPL | 2007 | Mesalazine, Infliximab | low-grade colitis with architectural defects in the epithelium and cryptabscesses | intermediate disease activity | n.a. | <5 | |
| UC 12 | 48/f | LPL | 2005 | Mesalazine | inconspicuous colon frame, proctitis | minimal disease activity | n.a. | n.a. | |
| UC13 | 26/f | LPL | 2019 | Mesalazine | inflammation in terminal Ileum, colon: inconspicuous | minimal disease activity | n.a. | n.a. | |
| UC14 | 55/f | LPL | 1985 | Sulfasalazine, Prednisolone | chronical low-grade colitis with ulcers and absesses in the crypts, cryptitis with architectural defects and general inflammatory epithelial changes | intermediate disease activity | n.a. | 124 | treatment with Abatacept (rheumatoid arthritis) |
| UC15 | 21/m | LPL | 2018 | Mesalazine, Infliximab | severe inflammatory changes in mucosa of the whole colon frame, fibrin coatings, some pseudopolyps | severe disease activity | n.a. | 3200 | |
S2 Table: Detailed cohort statistics of patients suffering from UC who donated colonic biopsies. UC, ulcerative colitis; LPL, lamina propria lymphocytes; LTX, patient with liver transplant; PSC, primarily sclerosing cholangitis; MMF, mycophenolate mofetil; AIH, autoimmune hepatitis.

## Slide 3
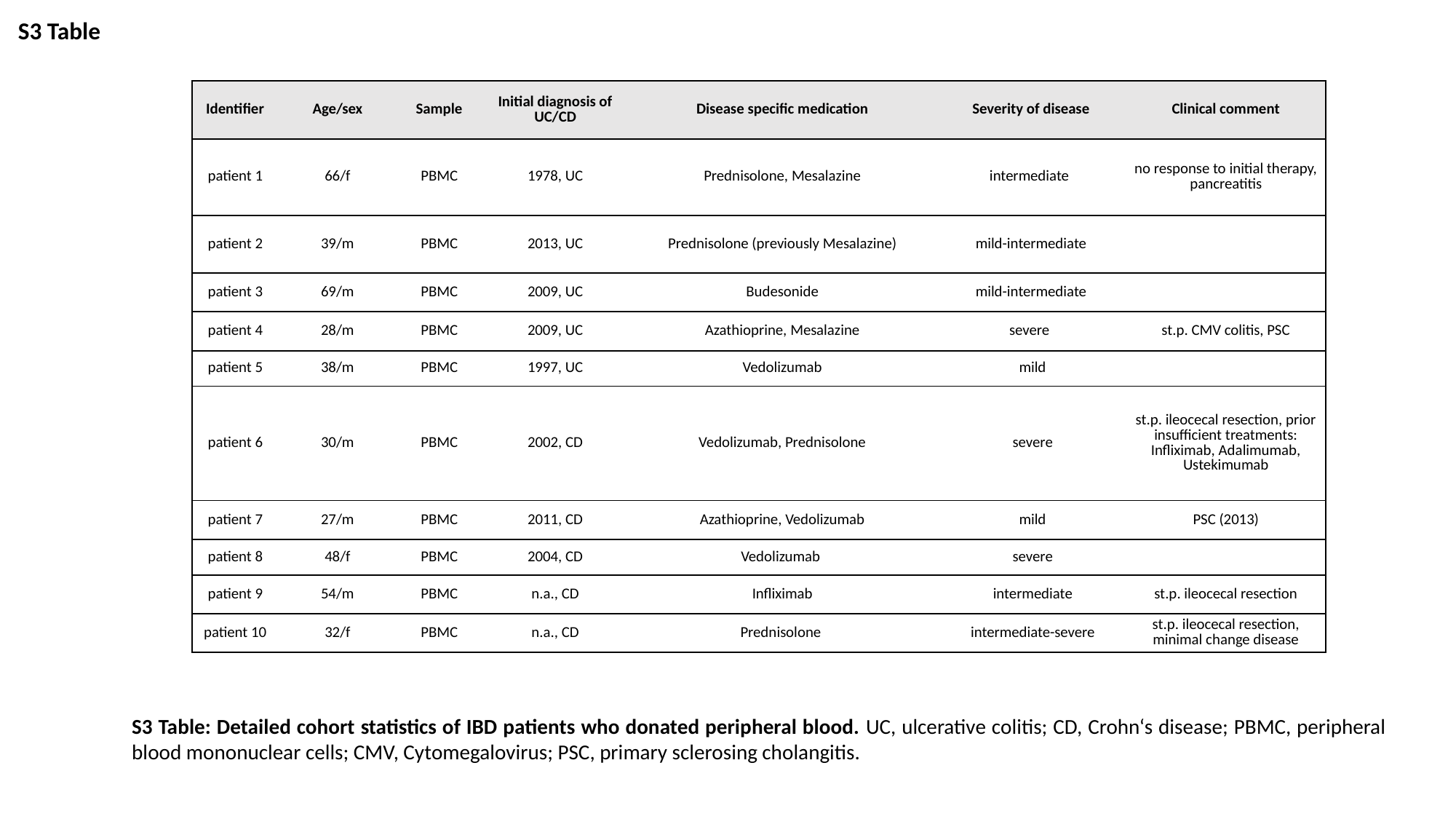

S3 Table
| Identifier | Age/sex | Sample | Initial diagnosis of UC/CD | Disease specific medication | Severity of disease | Clinical comment |
| --- | --- | --- | --- | --- | --- | --- |
| patient 1 | 66/f | PBMC | 1978, UC | Prednisolone, Mesalazine | intermediate | no response to initial therapy, pancreatitis |
| patient 2 | 39/m | PBMC | 2013, UC | Prednisolone (previously Mesalazine) | mild-intermediate | |
| patient 3 | 69/m | PBMC | 2009, UC | Budesonide | mild-intermediate | |
| patient 4 | 28/m | PBMC | 2009, UC | Azathioprine, Mesalazine | severe | st.p. CMV colitis, PSC |
| patient 5 | 38/m | PBMC | 1997, UC | Vedolizumab | mild | |
| patient 6 | 30/m | PBMC | 2002, CD | Vedolizumab, Prednisolone | severe | st.p. ileocecal resection, prior insufficient treatments: Infliximab, Adalimumab, Ustekimumab |
| patient 7 | 27/m | PBMC | 2011, CD | Azathioprine, Vedolizumab | mild | PSC (2013) |
| patient 8 | 48/f | PBMC | 2004, CD | Vedolizumab | severe | |
| patient 9 | 54/m | PBMC | n.a., CD | Infliximab | intermediate | st.p. ileocecal resection |
| patient 10 | 32/f | PBMC | n.a., CD | Prednisolone | intermediate-severe | st.p. ileocecal resection, minimal change disease |
S3 Table: Detailed cohort statistics of IBD patients who donated peripheral blood. UC, ulcerative colitis; CD, Crohn‘s disease; PBMC, peripheral blood mononuclear cells; CMV, Cytomegalovirus; PSC, primary sclerosing cholangitis.

## Slide 4
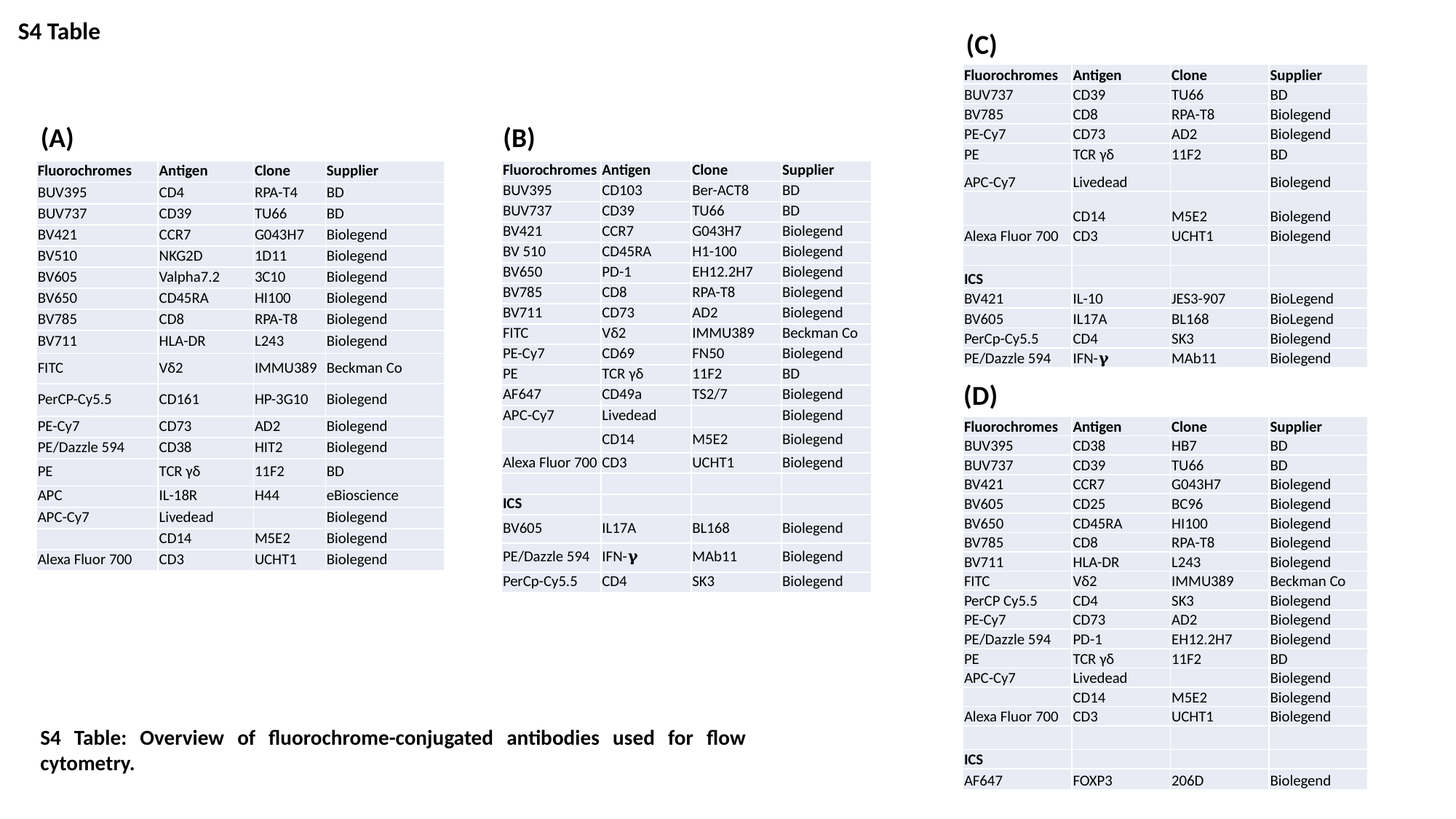

S4 Table
(C)
| Fluorochromes | Antigen | Clone | Supplier |
| --- | --- | --- | --- |
| BUV737 | CD39 | TU66 | BD |
| BV785 | CD8 | RPA-T8 | Biolegend |
| PE-Cy7 | CD73 | AD2 | Biolegend |
| PE | TCR γδ | 11F2 | BD |
| APC-Cy7 | Livedead | | Biolegend |
| | CD14 | M5E2 | Biolegend |
| Alexa Fluor 700 | CD3 | UCHT1 | Biolegend |
| | | | |
| ICS | | | |
| BV421 | IL-10 | JES3-907 | BioLegend |
| BV605 | IL17A | BL168 | BioLegend |
| PerCp-Cy5.5 | CD4 | SK3 | Biolegend |
| PE/Dazzle 594 | IFN-𝛄 | MAb11 | Biolegend |
(A)
(B)
| Fluorochromes | Antigen | Clone | Supplier |
| --- | --- | --- | --- |
| BUV395 | CD103 | Ber-ACT8 | BD |
| BUV737 | CD39 | TU66 | BD |
| BV421 | CCR7 | G043H7 | Biolegend |
| BV 510 | CD45RA | H1-100 | Biolegend |
| BV650 | PD-1 | EH12.2H7 | Biolegend |
| BV785 | CD8 | RPA-T8 | Biolegend |
| BV711 | CD73 | AD2 | Biolegend |
| FITC | Vδ2 | IMMU389 | Beckman Co |
| PE-Cy7 | CD69 | FN50 | Biolegend |
| PE | TCR γδ | 11F2 | BD |
| AF647 | CD49a | TS2/7 | Biolegend |
| APC-Cy7 | Livedead | | Biolegend |
| | CD14 | M5E2 | Biolegend |
| Alexa Fluor 700 | CD3 | UCHT1 | Biolegend |
| | | | |
| ICS | | | |
| BV605 | IL17A | BL168 | Biolegend |
| PE/Dazzle 594 | IFN-𝛄 | MAb11 | Biolegend |
| PerCp-Cy5.5 | CD4 | SK3 | Biolegend |
| Fluorochromes | Antigen | Clone | Supplier |
| --- | --- | --- | --- |
| BUV395 | CD4 | RPA-T4 | BD |
| BUV737 | CD39 | TU66 | BD |
| BV421 | CCR7 | G043H7 | Biolegend |
| BV510 | NKG2D | 1D11 | Biolegend |
| BV605 | Valpha7.2 | 3C10 | Biolegend |
| BV650 | CD45RA | HI100 | Biolegend |
| BV785 | CD8 | RPA-T8 | Biolegend |
| BV711 | HLA-DR | L243 | Biolegend |
| FITC | Vδ2 | IMMU389 | Beckman Co |
| PerCP-Cy5.5 | CD161 | HP-3G10 | Biolegend |
| PE-Cy7 | CD73 | AD2 | Biolegend |
| PE/Dazzle 594 | CD38 | HIT2 | Biolegend |
| PE | TCR γδ | 11F2 | BD |
| APC | IL-18R | H44 | eBioscience |
| APC-Cy7 | Livedead | | Biolegend |
| | CD14 | M5E2 | Biolegend |
| Alexa Fluor 700 | CD3 | UCHT1 | Biolegend |
(D)
| Fluorochromes | Antigen | Clone | Supplier |
| --- | --- | --- | --- |
| BUV395 | CD38 | HB7 | BD |
| BUV737 | CD39 | TU66 | BD |
| BV421 | CCR7 | G043H7 | Biolegend |
| BV605 | CD25 | BC96 | Biolegend |
| BV650 | CD45RA | HI100 | Biolegend |
| BV785 | CD8 | RPA-T8 | Biolegend |
| BV711 | HLA-DR | L243 | Biolegend |
| FITC | Vδ2 | IMMU389 | Beckman Co |
| PerCP Cy5.5 | CD4 | SK3 | Biolegend |
| PE-Cy7 | CD73 | AD2 | Biolegend |
| PE/Dazzle 594 | PD-1 | EH12.2H7 | Biolegend |
| PE | TCR γδ | 11F2 | BD |
| APC-Cy7 | Livedead | | Biolegend |
| | CD14 | M5E2 | Biolegend |
| Alexa Fluor 700 | CD3 | UCHT1 | Biolegend |
| | | | |
| ICS | | | |
| AF647 | FOXP3 | 206D | Biolegend |
S4 Table: Overview of fluorochrome-conjugated antibodies used for flow cytometry.

## Slide 5
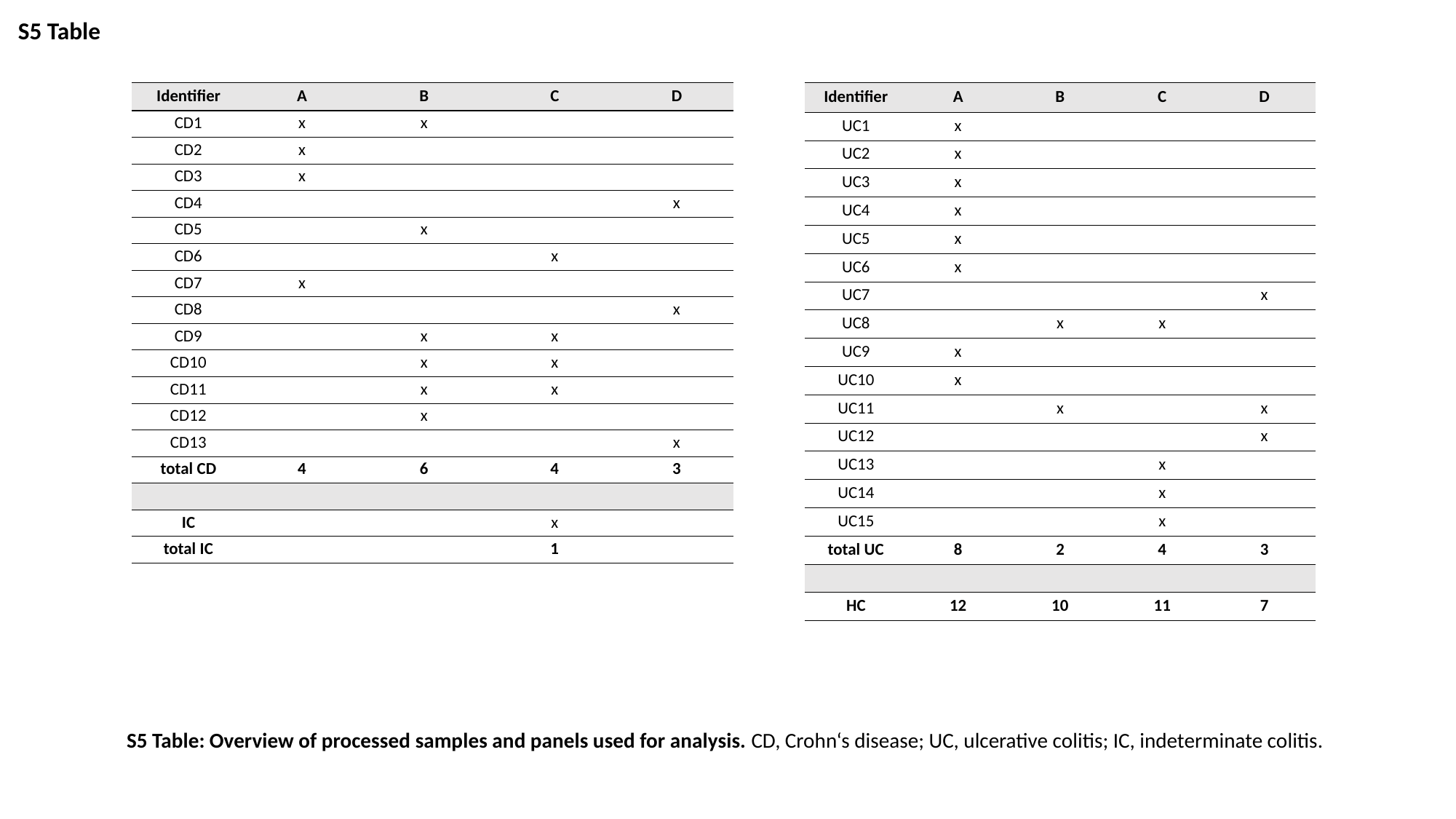

S5 Table
| Identifier | A | B | C | D |
| --- | --- | --- | --- | --- |
| CD1 | x | x | | |
| CD2 | x | | | |
| CD3 | x | | | |
| CD4 | | | | x |
| CD5 | | x | | |
| CD6 | | | x | |
| CD7 | x | | | |
| CD8 | | | | x |
| CD9 | | x | x | |
| CD10 | | x | x | |
| CD11 | | x | x | |
| CD12 | | x | | |
| CD13 | | | | x |
| total CD | 4 | 6 | 4 | 3 |
| | | | | |
| IC | | | x | |
| total IC | | | 1 | |
| Identifier | A | B | C | D |
| --- | --- | --- | --- | --- |
| UC1 | x | | | |
| UC2 | x | | | |
| UC3 | x | | | |
| UC4 | x | | | |
| UC5 | x | | | |
| UC6 | x | | | |
| UC7 | | | | x |
| UC8 | | x | x | |
| UC9 | x | | | |
| UC10 | x | | | |
| UC11 | | x | | x |
| UC12 | | | | x |
| UC13 | | | x | |
| UC14 | | | x | |
| UC15 | | | x | |
| total UC | 8 | 2 | 4 | 3 |
| | | | | |
| HC | 12 | 10 | 11 | 7 |
S5 Table: Overview of processed samples and panels used for analysis. CD, Crohn‘s disease; UC, ulcerative colitis; IC, indeterminate colitis.

## Slide 6
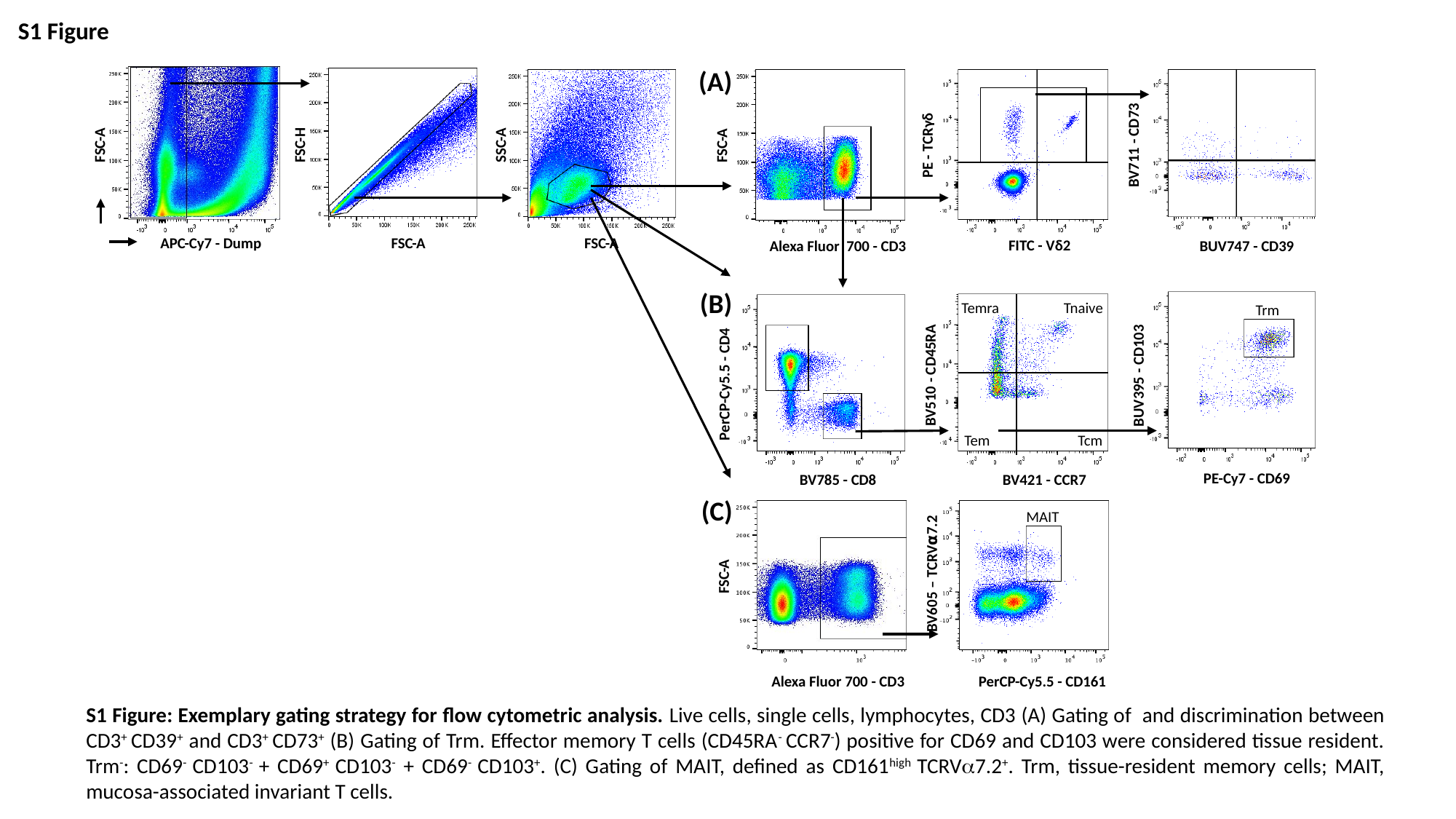

S1 Figure
(A)
BV711 - CD73
PE - TCRγδ
FSC-A
SSC-A
FSC-A
FSC-H
APC-Cy7 - Dump
FSC-A
FSC-A
FITC - Vδ2
BUV747 - CD39
Alexa Fluor 700 - CD3
(B)
Tnaive
Temra
Trm
BV510 - CD45RA
BUV395 - CD103
PerCP-Cy5.5 - CD4
Tem
Tcm
PE-Cy7 - CD69
BV785 - CD8
BV421 - CCR7
(C)
MAIT
BV605 – TCRV⍺7.2
FSC-A
Alexa Fluor 700 - CD3
PerCP-Cy5.5 - CD161

## Slide 7
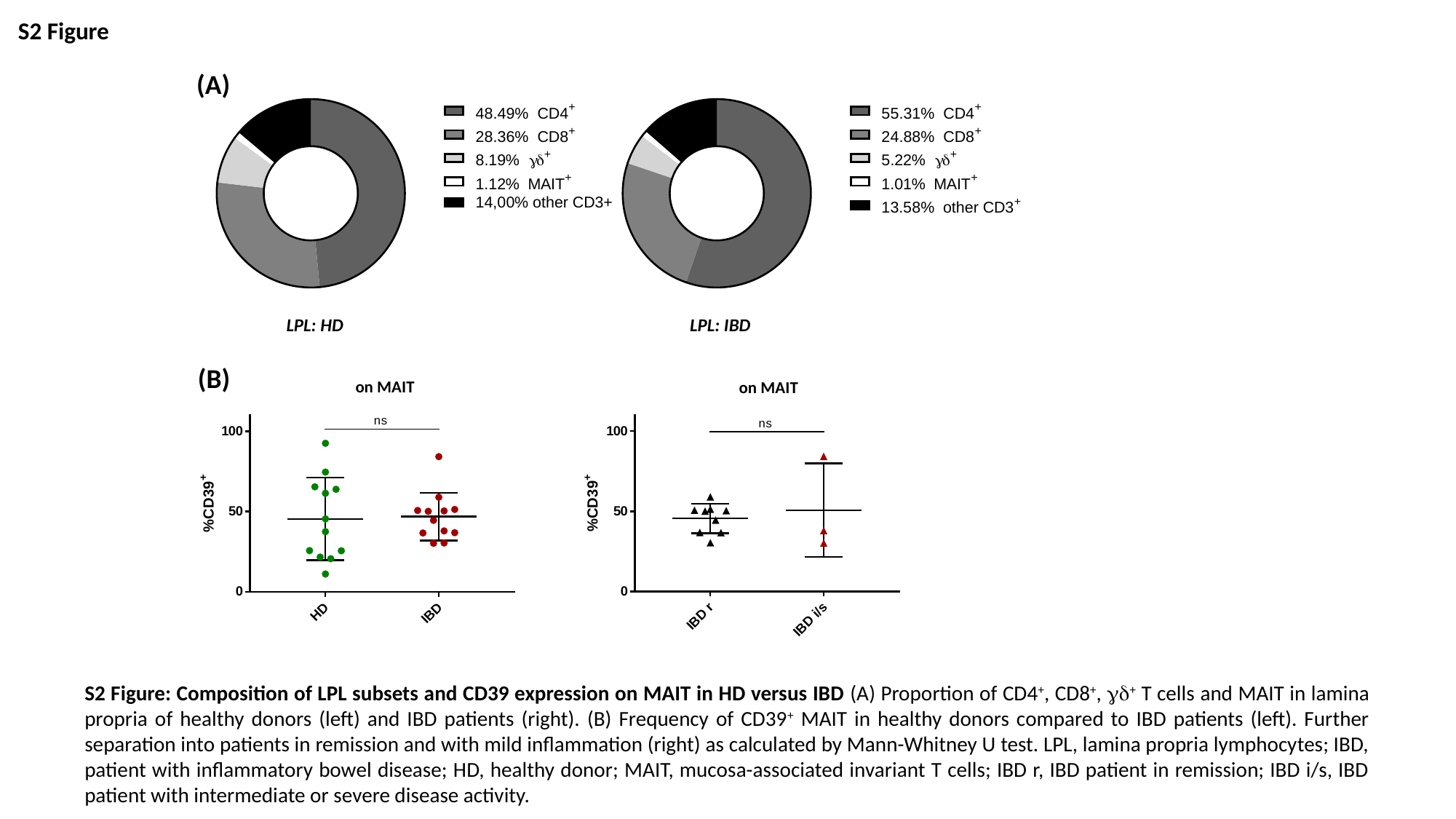

S2 Figure
(A)
LPL: HD
LPL: IBD
(B)
on MAIT
on MAIT
S2 Figure: Composition of LPL subsets and CD39 expression on MAIT in HD versus IBD (A) Proportion of CD4+, CD8+, + T cells and MAIT in lamina propria of healthy donors (left) and IBD patients (right). (B) Frequency of CD39+ MAIT in healthy donors compared to IBD patients (left). Further separation into patients in remission and with mild inflammation (right) as calculated by Mann-Whitney U test. LPL, lamina propria lymphocytes; IBD, patient with inflammatory bowel disease; HD, healthy donor; MAIT, mucosa-associated invariant T cells; IBD r, IBD patient in remission; IBD i/s, IBD patient with intermediate or severe disease activity.

## Slide 8
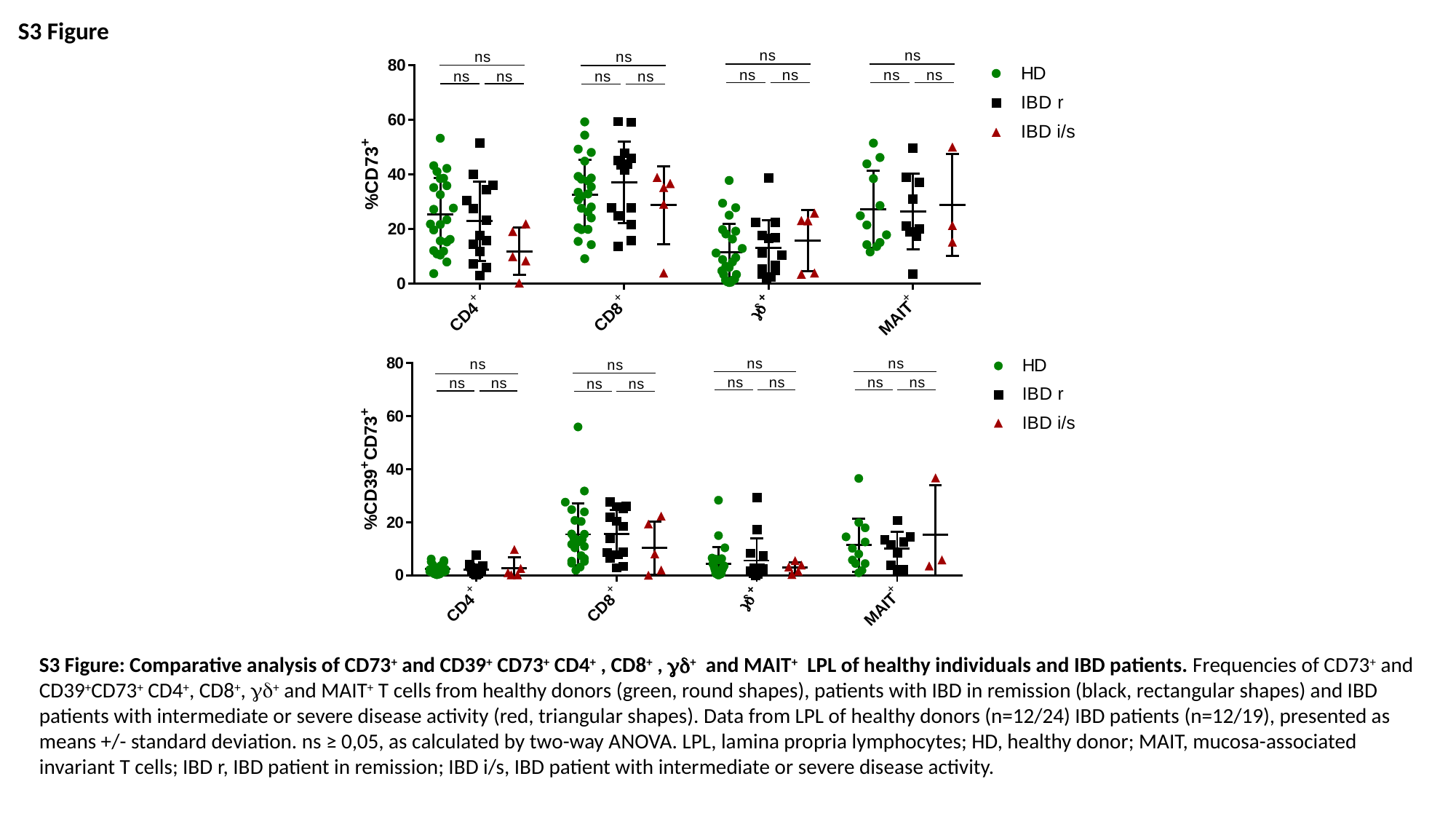

S3 Figure
S3 Figure: Comparative analysis of CD73+ and CD39+ CD73+ CD4+ , CD8+ , + and MAIT+ LPL of healthy individuals and IBD patients. Frequencies of CD73+ and CD39+CD73+ CD4+, CD8+, + and MAIT+ T cells from healthy donors (green, round shapes), patients with IBD in remission (black, rectangular shapes) and IBD patients with intermediate or severe disease activity (red, triangular shapes). Data from LPL of healthy donors (n=12/24) IBD patients (n=12/19), presented as means +/- standard deviation. ns ≥ 0,05, as calculated by two-way ANOVA. LPL, lamina propria lymphocytes; HD, healthy donor; MAIT, mucosa-associated invariant T cells; IBD r, IBD patient in remission; IBD i/s, IBD patient with intermediate or severe disease activity.

## Slide 9
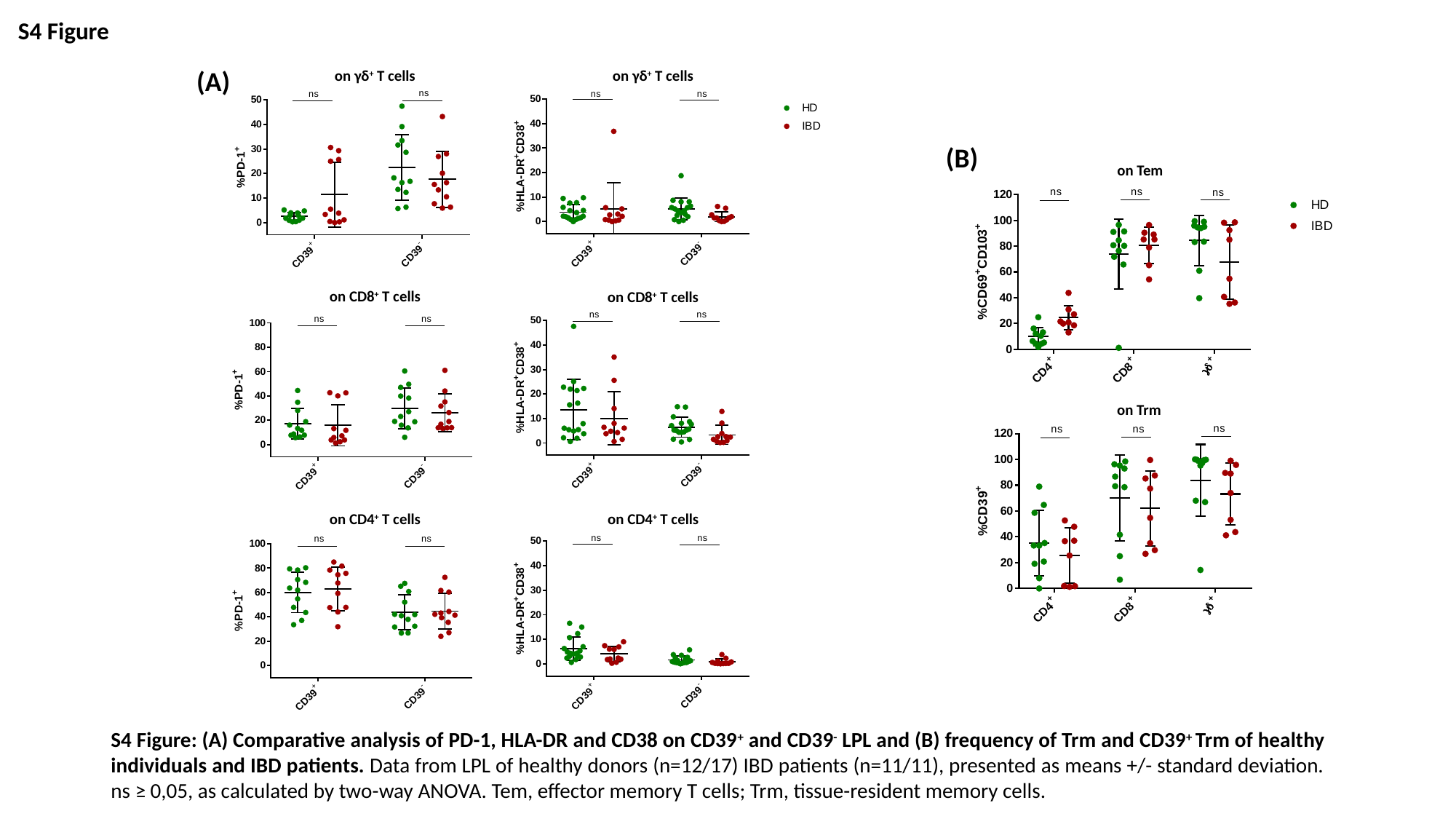

S4 Figure
(A)
on γδ+ T cells
on γδ+ T cells
(B)
on Tem
on CD8+ T cells
on CD8+ T cells
on Trm
on CD4+ T cells
on CD4+ T cells
S4 Figure: (A) Comparative analysis of PD-1, HLA-DR and CD38 on CD39+ and CD39- LPL and (B) frequency of Trm and CD39+ Trm of healthy individuals and IBD patients. Data from LPL of healthy donors (n=12/17) IBD patients (n=11/11), presented as means +/- standard deviation. ns ≥ 0,05, as calculated by two-way ANOVA. Tem, effector memory T cells; Trm, tissue-resident memory cells.

## Slide 10
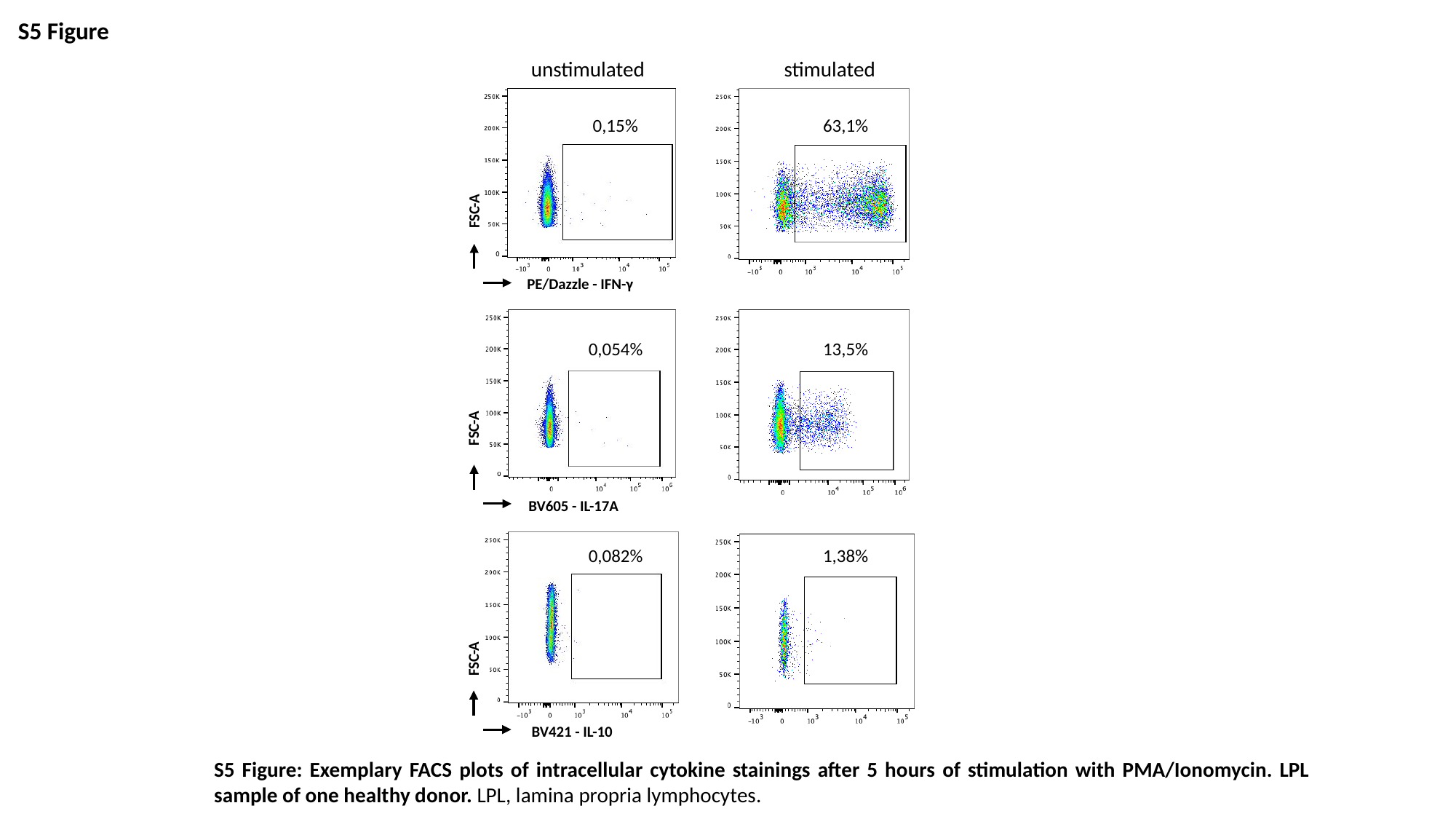

S5 Figure
unstimulated
stimulated
0,15%
63,1%
FSC-A
PE/Dazzle - IFN-γ
0,054%
13,5%
FSC-A
BV605 - IL-17A
0,082%
1,38%
FSC-A
BV421 - IL-10
S5 Figure: Exemplary FACS plots of intracellular cytokine stainings after 5 hours of stimulation with PMA/Ionomycin. LPL sample of one healthy donor. LPL, lamina propria lymphocytes.

## Slide 11
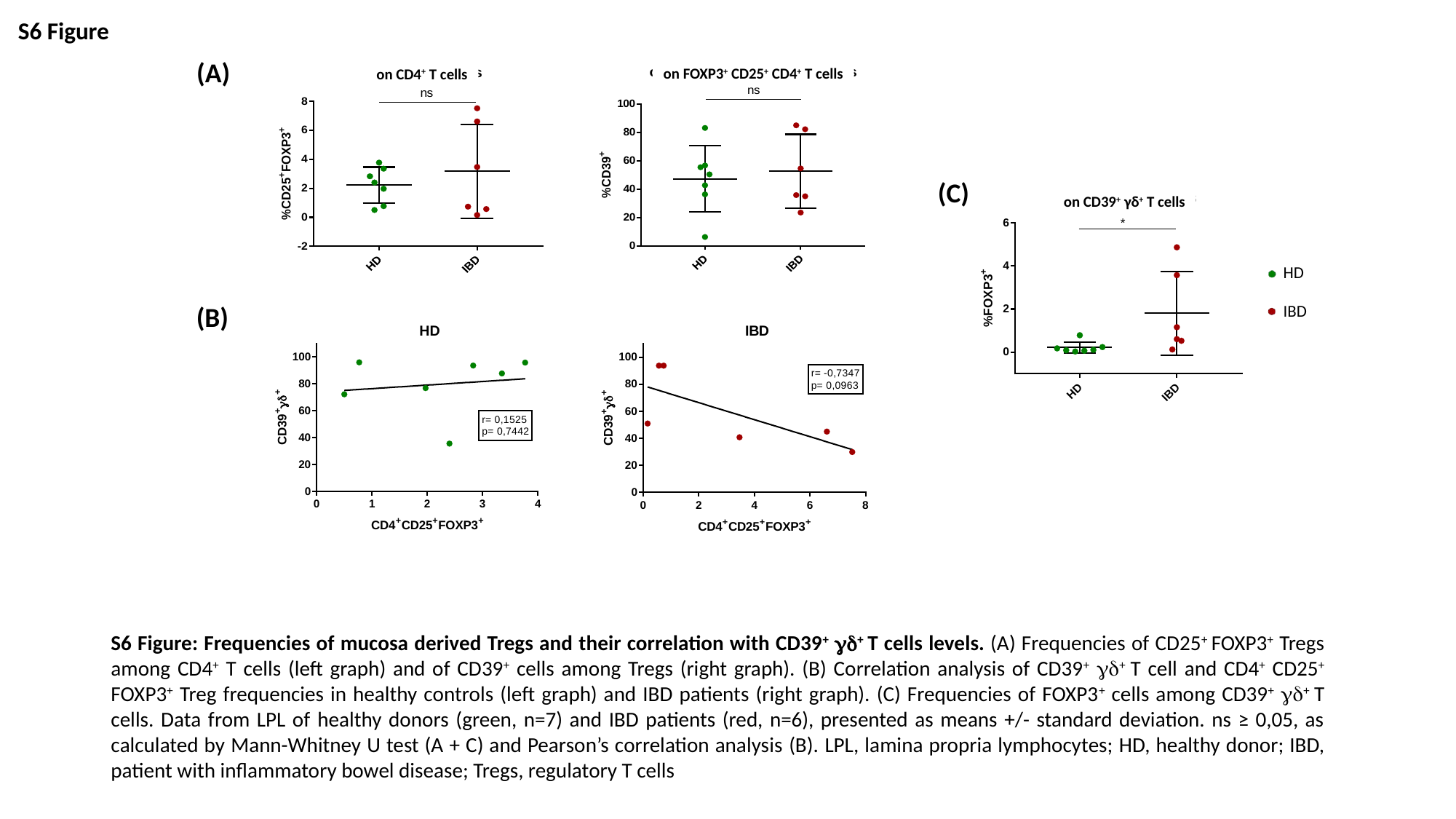

S6 Figure
(A)
on FOXP3+ CD25+ CD4+ T cells
on CD4+ T cells
(C)
on CD39+ γδ+ T cells
HD
(B)
IBD
S6 Figure: Frequencies of mucosa derived Tregs and their correlation with CD39+ + T cells levels. (A) Frequencies of CD25+ FOXP3+ Tregs among CD4+ T cells (left graph) and of CD39+ cells among Tregs (right graph). (B) Correlation analysis of CD39+ + T cell and CD4+ CD25+ FOXP3+ Treg frequencies in healthy controls (left graph) and IBD patients (right graph). (C) Frequencies of FOXP3+ cells among CD39+ + T cells. Data from LPL of healthy donors (green, n=7) and IBD patients (red, n=6), presented as means +/- standard deviation. ns ≥ 0,05, as calculated by Mann-Whitney U test (A + C) and Pearson’s correlation analysis (B). LPL, lamina propria lymphocytes; HD, healthy donor; IBD, patient with inflammatory bowel disease; Tregs, regulatory T cells
